# Enhanced Optical Biosensing by Aerotaxy Ga(As)P Nanowire Platforms Suitable for Scalable Production

**DOI:** 10.1021/acsanm.2c01372

**Published:** 2022-07-01

**Authors:** Julia Valderas-Gutiérrez, Rubina Davtyan, Sudhakar Sivakumar, Nicklas Anttu, Yuyu Li, Patrick Flatt, Jae Yen Shin, Christelle N. Prinz, Fredrik Höök, Thoas Fioretos, Martin H. Magnusson, Heiner Linke

**Affiliations:** † NanoLund, Lund University, P.O. Box 118, SE-22100 Lund, Sweden; ‡ Division of Solid State Physics, Lund University, P.O. Box 118, SE-22100 Lund, Sweden; § Physics, Faculty of Science and Engineering, Åbo Akademi University, Henrikinkatu 2, FI-20500 Turku, Finland; ∥ AlignedBio AB, Medicon Village, Scheeletorget 1, SE-22363, Lund 22100, Sweden; ⊥ Department of Physics, Chalmers University of Technology, SE-41296 Göteborg, Sweden; # Division of Clinical Genetics, Lund University, SE-22185 Lund, Sweden

**Keywords:** semiconductor nanowires, aerotaxy, biosensing, lightguiding, scalable production

## Abstract

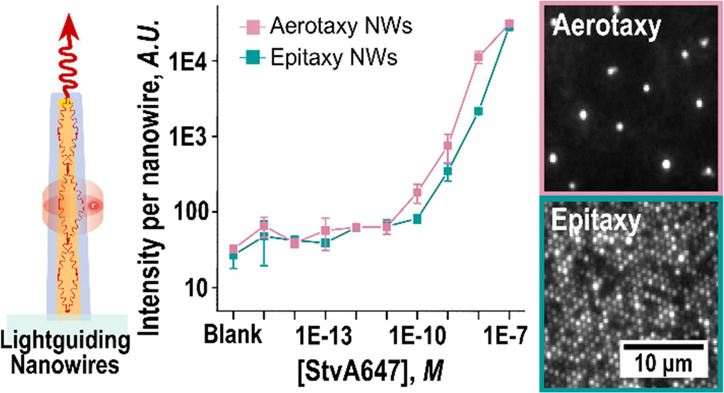

Sensitive detection of low-abundance biomolecules is central for diagnostic applications. Semiconductor nanowires can be designed to enhance the fluorescence signal from surface-bound molecules, prospectively improving the limit of optical detection. However, to achieve the desired control of physical dimensions and material properties, one currently uses relatively expensive substrates and slow epitaxy techniques. An alternative approach is aerotaxy, a high-throughput and substrate-free production technique for high-quality semiconductor nanowires. Here, we compare the optical sensing performance of custom-grown aerotaxy-produced Ga(As)P nanowires vertically aligned on a polymer substrate to GaP nanowires batch-produced by epitaxy on GaP substrates. We find that signal enhancement by individual aerotaxy nanowires is comparable to that from epitaxy nanowires and present evidence of single-molecule detection. Platforms based on both types of nanowires show substantially higher normalized-to-blank signal intensity than planar glass surfaces, with the epitaxy platforms performing somewhat better, owing to a higher density of nanowires. With further optimization, aerotaxy nanowires thus offer a pathway to scalable, low-cost production of highly sensitive nanowire-based platforms for optical biosensing applications.

## 1 Introduction

Fluorescence-based detection assays for biomolecules such as proteins, DNA or RNA, are essential tools in diagnosis, therapy, and monitoring of disease^1−5^ as well as in investigating fundamental biological processes.^6−8^ The ability to measure a signal arising from fluorescent molecules offers a low limit of detection (LOD) owing to high specificity and highly sensitive optical readout platforms.^9−13^ To this end, nanomaterials^14^ such as nanoparticles^15,16^ and quantum dots^16−18^ can be used as inorganic fluorescent probes for improved detection, and a number of approaches to enhanced biosensing have been presented, for example, the use of carbon-based nanoquenchers^19,20^ metal-enhanced fluorescence^21−23^ or plasmonic nanorods,^24^ among others.

**Figure 1:**
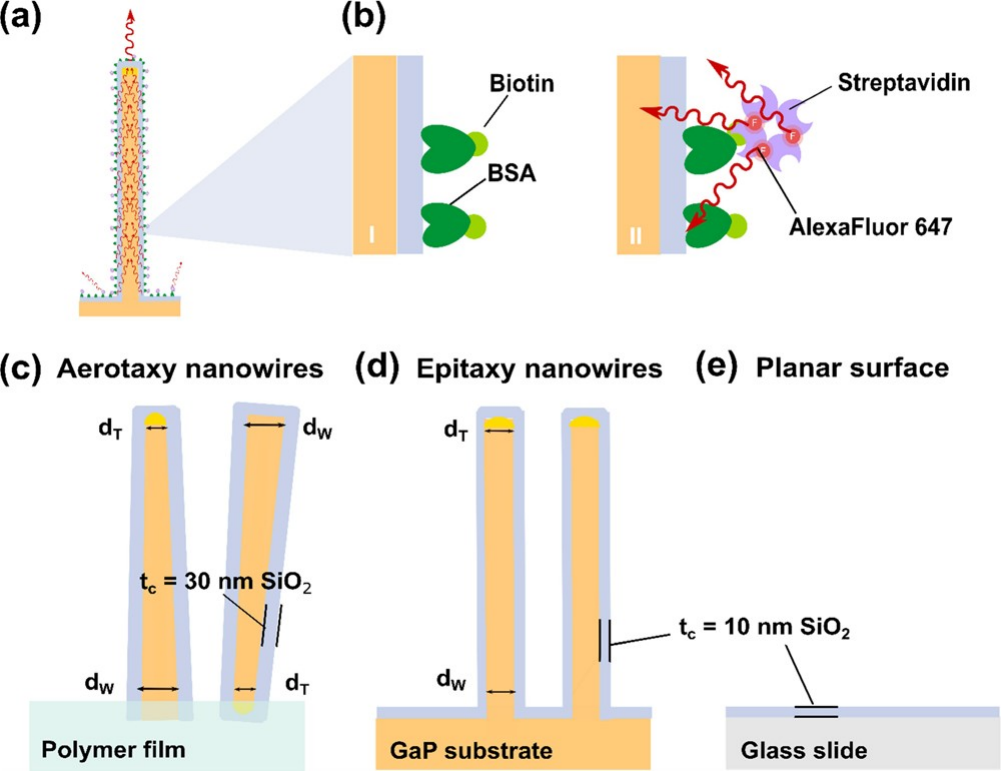
(a) Schematic representation of a single, vertical nanowire, illustrating the lightguiding effect which occurs when light emitted by a fluorophore bound to the nanowire surface is collected and re-emitted at the tip. (b) To functionalize the samples, biotin-conjugated bovine serum albumin (bBSA) is adsorbed to the SiO_2_ layer covering the nanowire platforms or the glass, respectively (I). Then, Alexa Fluor 647-labeled streptavidin (StvA647) binds with high specificity to biotin (II). The three sensing surfaces present different morphological and surface properties: (c) aerotaxy Ga(As)P nanowires, with a SiO_2_ coating of thickness *t*_c_ ≈ 30 nm, embedded in a polymer film that is not coated with SiO_2_. The aerotaxy nanowires are tapered, with an average diameter of *d*_T_ ≈ 90 nm at their thin end and *d*_W_ ≈ 137 nm at their wide end. The orientation of aerotaxy nanowires varies randomly, and the wide end of each individual nanowire can be either at the top or the bottom. (d) Epitaxy GaP nanowires have an average *d*_T_ ≈ 98 nm at the tip and average *d*_W_ ≈ 104 nm at the base and are grown on a GaP substrate, with a SiO_2_ layer (*t*_c_ ≈ 10 nm) on nanowires and substrates. (e) Planar glass surface, with a SiO_2_ layer (*t*_c_ ≈ 10 nm). (More details are shown in Table 1).

Another emerging and promising approach is the use of vertical semiconductor nanowires of high refractive index to enhance the signal from fluorescent molecules bound to the nanowire surface by more than one order of magnitude compared to flat surfaces without nanowires.^25,26^ This approach enables single-molecule detection even with conventional epifluorescence setups.^27^ The observed fluorescence enhancement is understood as the combination of several optical phenomena^28^ that are specific to high refractive index materials (Figure 1a): (i) nanowires of appropriately chosen diameter (typically around 100–150 nm, depending on refractive index of the nanowire material and fluorescence wavelength of the marker)^29^ can support waveguiding modes that collect light from surface-bound fluorophores and guide it to the nanowire tip (lightguiding effect);^30^ (ii) emission from the tip is expected to be directional,^31−33^ enabling effective collection with conventional microscopes, and (iii) the excitation of fluorophores is enhanced if bound sufficiently close to the nanowire surface.^34^

Lightguiding of fluorescent signals has been observed in a variety of nanowire structures with a strong dependence on their morphology and material:^29,35^ zinc oxide (ZnO) nanowires have shown signal enhancement for detection of proteins and DNA when compared to non-lightguiding platforms^36^ and have displayed promising results for protein detection^37^ including disease biomarkers,^38,39^ even surpassing ELISA performance.^40^ Among III–V semiconductors, lightguiding has been described for various materials, for example, indium arsenide (InAs),^34,35^ gallium arsenide (GaAs),^41^ and gallium phosphide (GaP).^30^

To date, GaP nanowires appear to be a particularly adequate choice for use in optical detection of fluorescence, given their high refractive index,^42^ their transparency due to the absence of a direct band gap in the visible range,^43^ and their biocompatibility;^44−47^ and their lightguiding has been characterized as a function of the fluorophore wavelength and their geometry.^29^ Previous studies demonstrated the applicability of GaP nanowire arrays in single-molecule detection to study the diffusivity and concentration of membrane-bound proteins,^27^ and they have been used for the detection of protein biomarkers, providing a 20-fold increase on sensitivity in contrast to conventional flat sensing surfaces.^26^ In addition, vertically oriented GaP nanowires can be synthesized on GaP substrates by metal–organic vapor phase epitaxy (MOVPE), a nanoparticle-seeded, vapor–liquid–solid process that allows for optimization of fluorescence enhancement with much higher control over morphology, orientation, and positioning, as compared to solution-based methods used for instance for ZnO nanowires. However, MOVPE of GaP nanowires is not easily compatible with large-scale biosensing applications, for example, point-of-care diagnostics, because nanowire growth is a slow process and requires expensive GaP wafer substrates.^48^

A high-throughput alternative to epitaxy for controlled nanowire synthesis is the use of aerotaxy. Aerotaxy is an aerosol-based, continuous-flow method for synthesis of nanowires that does not require any growth substrate such as a wafer. Furthermore, aerotaxy takes place at near-atmospheric pressure, allowing growth rates that can be ∼1000 times higher than substrate-based methods (∼1 μm/s), enabling the production of large amounts of nanowires (1 mg/h in our current reactor, scalable to grams).^49^ Importantly, in recent years, high control over optical, structural, and geometric properties of aerotaxy nanowires has been established,^50−54^ opening the door to high-performance applications (photovoltaics, photocatalysis, or sensing). Nanowires exit the aerotaxy process as an aerosol, to be filtered and collected.^55^ They initially display a random orientation, but a proprietary alignment technology exists to present the nanowires in an aligned, vertical orientation suitable for optical biosensing.^56,57^

Here, we demonstrate the use of such aerotaxy-produced nanowires for highly sensitive optical biosensing. We developed the aerotaxy growth protocol^50,54^ for Ga(As)P nanowires with an average diameter of 110 nm, chosen for displaying optimal lightguiding properties^29^ at the wavelength of the fluorophore used in this work, in which the fundamental HE_11_ waveguide mode leads to a strong in-coupling of light. For their applicability as biosensors, we have implemented an assay based on fluorescently labeled proteins (Figure 1a,b) to investigate the lightguiding effect of platforms containing aerotaxy nanowires (Figure 1c) and to benchmark them against epitaxy nanowires (Figure 1d). We also include a planar glass platform without nanowires as a control (Figure 1e), allowing us to compare the sensing capability of aerotaxy Ga(As)P nanowires to epitaxy-based GaP nanowires and to planar glass surfaces. All three types of platforms were coated with a layer of SiO_2_ to ensure equivalent surface chemistry. By extracting the total (*I*_S_) and relative (normalized to a blank without analyte, *I*_N_) signal intensity after protein binding, our results show an improvement on both parameters by one and two orders of magnitude for aerotaxy and epitaxy nanowire platforms, respectively, when compared to the planar surface control. Importantly, both nanowire platforms displayed comparable signal enhancement for individual nanowires. In particular, single-molecule detection was verified for both types of substrates by correlating the number of bright nanowires with the brightness of individual nanowires as the analyte concentration was increased. We conclude that aerotaxy is a fully viable method for the synthesis of nanowires for high-sensitivity, bioanalytical sensing applications.

## 2 Experimental Section

Based on optics modeling for optimal lightguiding, the nanowire platforms were designed with the aim to display arrays of vertical nanowires with a diameter of ∼110 nm, expected to provide good lightguiding at the chosen fluorophore wavelength,^29^ and with a spacing of 1 μm or more, sufficient to avoid optical nanowire–nanowire overlap at the illumination wavelength of 650 nm,^26^ enabling single-nanowire analysis. In addition, for biomolecular functionalization purposes, the nanowires were modified with a SiO_2_ coating of thickness *t*_c_.

### 2.1 Nanowire Synthesis

**Figure 2:**
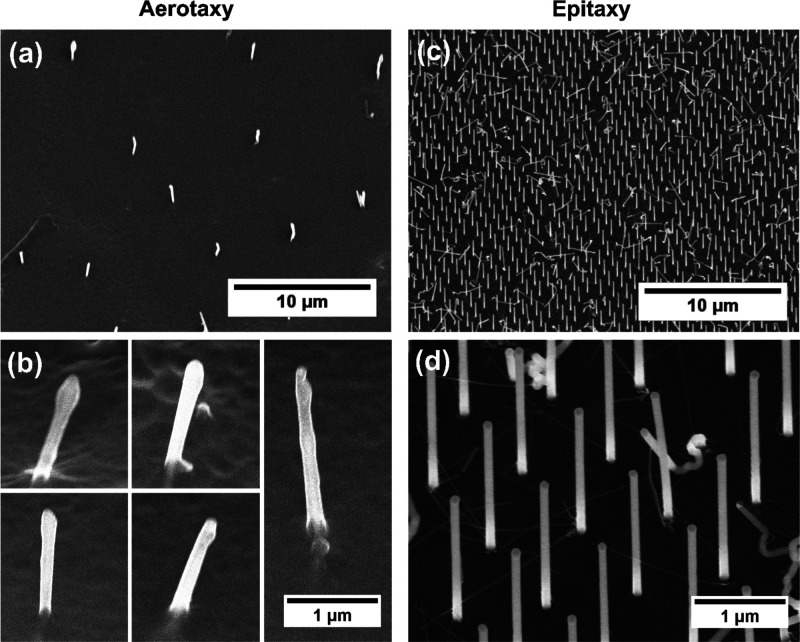
(a) Representative scanning electron microscopy (SEM) images of aerotaxy Ga(As)P nanowires (stage tilt 30°) (b) and close-up view on several individual aerotaxy nanowires from different regions, illustrating the heterogeneity in terms of morphology and orientation. (c) Epitaxy GaP nanowires (stage tilt 30°) and (d) close-up view of some epitaxy nanowires. Even though 30% of the epitaxy nanowires are kinked or defective, the remaining 70% exhibit a good homogeneity in morphology, orientation, and pitch.

For the aerotaxy Ga(As)P nanowire growth, an aerosol of Au particles with diameter 50 ± 15 nm was mixed with trimethylgallium (TMGa) to form an Au–Ga alloy in an aerotaxy reactor.^52^ When exposed to phosphine (PH_3_), supersaturation and nucleation of new atomic planes occurs, leading to preferential nanowire growth in the III–V (111)B direction.^50,54^ The content of As (GaAs_*x*_P_1–*x*,_*x* ≈ 0.2) originated from residual As on the inner walls of the aerotaxy reactor, a memory effect due to previous growths of GaAs nanowires.^58^ Aerotaxy nanowires showed a tapering factor of ∼0.2, calculated as (*d*_W_ – *d*_T_)/(*d*_W_ + *d*_T_), with an end-to-end diameter variation from ∼90 to ∼140 nm, and the presence of occasional irregularities or “lumps” of material (Figure 2a,b).

Epitaxy GaP nanowires were grown by MOVPE from 107 ± 8 nm Au nanoparticles on (111)B GaP substrates, deposited using displacement Talbot lithography (DTL),^59−61^ guided by a SiN mask to achieve a periodic disposition of nanowires with a regular spacing of ∼1 μm and a density of 1.19 NWs/μm^2^ (∼30% of nanowires were estimated to be kinked or defective). The tapering factor was ∼0.02, with an end-to-end diameter variation from ∼98 to ∼104 nm (Figure 2c,d). Additional details on nanowire properties of all nanowires used for both growth techniques are shown in Table 1.

**Table 1:** Morphological Characterization of the Nanowire Platforms Involved in This Study^a^

	aerotaxy nanowires	epitaxy nanowires
*d*_W_ (nm)	137 ± 21	104 ± 6
*d*_T_ (nm)	89 ± 21	98 ± 6
average diameter (nm)	113 ± 15	101 ± 4
length, *L* (μm)	2.23 ± 0.39	2.87 ± 0.51
*t*_c_ of SiO_2_ (nm)	32 ± 6	11 ± 1
spacing (μm)	≈5–10	0.99
density (nanowires/μm^2^)	0.018	0.860 (considering only the non-kinked nanowires, 72% of the total density of 1.19)
tapering factor	0.217 ± 0.107	0.024 ± 0.016
tilt away from vertical (degrees)	0–15	negligible for non-kinked nanowires
lateral surface area per nanowire (μm^2^/nanowire)	1.25 ± 0.18	1.10 ± 0.14
effective nanowire surface area per platform area (μm^2^/μm^2^)	0.022 ± 0.003	0.95 ± 0.13

a *d*_W_, *d*_T_, *L*, and *t*_c_ were measured using SEM images for *N* ≥ 30 nanowires. The average diameter was calculated as (*d*_W_ + *d*_T_)/2. For aerotaxy films, *L* was measured on horizontal nanowires before being embedded into the polymer film. Spacing between nanowires in epitaxy samples was achieved by using a specific mask for DTL. Density values were determined from optical microscopy images for aerotaxy and SEM images for epitaxy (*N* ≥ 600). In the case of epitaxy platforms, we consider the density of non-kinked nanowires since they are the only ones whose signal can be detected. The tapering factor (*N* ≥ 30) is calculated as (*d*_W_ – *d*_T_)/(*d*_W_ + *d*_T_). The lateral surface area per nanowire was calculated according to the formula for a truncated cone:  The effective surface area is based on the surface area per nanowire multiplied by the density of nanowires.

### 2.2 Platform Preparation

Using a proprietary technology (AlignedBio AB, Lund, Sweden), aerotaxy nanowires were coated before alignment with a SiO_2_ layer of *t*_c_ ≈ 30 nm (minimum thickness currently achievable, inherent to aerotaxy fabrication process) and randomly deposited vertically on uncoated polymer films. The nanowires were partially embedded into this substrate with approximately 2 μm of the nanowire protruding from the polymer. As a consequence of the deposition process, the exposed length and the orientation varied between nanowires, and they tilt away from the perfect vertical direction in a range from 0 to 15° (Figure 2a,b).

Epitaxy-grown GaP nanowires and substrates were coated with a SiO_2_ layer of *t*_c_ ≈ 10 nm (optimal thickness to ensure both a thin oxide for the highest performance in terms of lightguiding and reliable oxide coating), using atomic layer deposition (ALD). (See Supporting Information Section 1 for further details on nanowire growth and platform processing).

The planar platforms were conventional glass coverslips (25 × 60 mm^2^ #1 from Menzel-Gläser, ThermoFisher Scientific, USA) coated with a SiO_2_ layer (*t*_c_ ≈ 10 nm) using ALD.

Further scanning electron microscopy (SEM) and transmission electron microscopy–energy-dispersive spectroscopy (TEM–EDS) characterization of the aerotaxy nanowires relevant for this work is included on the Supporting Information Section 2.

### 2.3 Sample Functionalization

Both aerotaxy and epitaxy nanowire platforms were attached to the ceiling of the same 30 μL channel of a μ-Slide VI 0.4 microfluidic flow chamber (ibidi, Germany) (see Supporting Information Section 3 for further details on this flow chamber) using a double-sided adhesive transfer tape 467MP (3M, USA) and subsequently hermetically closed by the SiO_2_-coated glass slide, which also serves as the planar surface evaluated in this work. Thereafter, a well-established streptavidin-biotin biorecognition assay^62^ was simultaneously performed for all three surfaces. First, a solution of 6 μM of biotinylated bovine serum albumin (bBSA, Sigma-Aldrich, USA) in phosphate-buffered saline buffer (PBS) (pH 7.2, 1× w/o Ca^2+^ or Mg^2+^, from ThermoFisher Scientific, USA) was added (115 μL/channel) to accomplish complete coverage of the surface area, given the strong absorption of BSA on hydrophilic SiO_2_ surfaces.^63,64^ The samples were incubated for 1 h at room temperature (RT) with agitation in a rocker shaker. The surfaces were then rinsed with PBS (120 μL/channel, ×3) followed by incubation with solutions containing streptavidin labeled with Alexa Fluor 647 (StvA647, ThermoFisher Scientific, USA) in PBS (150 μL/channel) at different concentrations (9 concentrations prepared as 10-fold serial dilutions from 100 nM to 1 fM plus a blank without StvA647). Subsequent incubation was performed protected from light for 1 h at RT and with agitation. Finally, each channel was washed with PBS (120 μL/channel, ×3).

### 2.4 Image Acquisition

Within 24 h after incubation and washing, the samples were imaged in epifluorescence with an inverted microscope (Eclipse T2000-U, Nikon, Japan), using a 60×, 1 NA, water-dipping objective, a Chroma 49006 ET-Cy5 filter cube (both from Nikon, Japan), and an iXon Life 897 EMCCD camera (Andor, Oxford Instruments, UK). Electron-multiplying gain was set to 200 and the exposure time to 200 milliseconds, acquiring stacks of 25 time-lapse frames, projected into a single image of averaged intensity. A 640 nm laser was used as an illumination source, kept at ∼30 mW (30% of maximum power) during acquisition.

### 2.5 Image Analysis

At least 10 images from different regions were acquired per sample and experimental condition, and each of them was cropped into a 200 × 200 pixels (53.3 × 53.3 μm^2^) region of interest (ROI) of homogeneous laser illumination, using ImageJ-Fiji software.^65^

**Figure 3:**
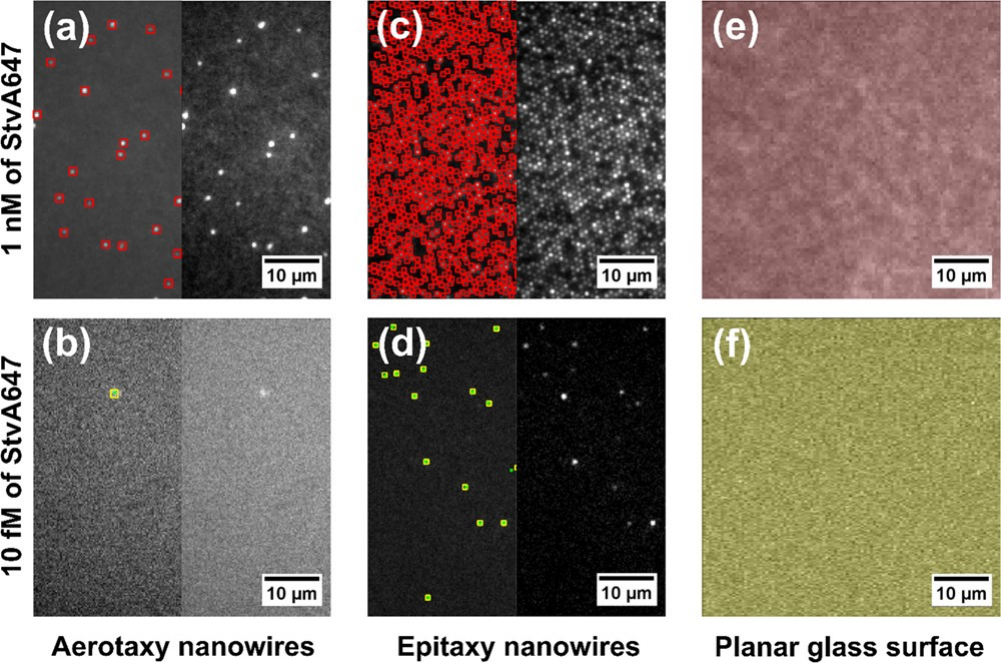
Examples of image analysis for the calculations of fluorescence intensity on ROIs of the three evaluated surfaces, for high (top row) and low (bottom row) concentrations of StvA647, respectively. (a,b) For aerotaxy Ga(As)P nanowire platforms, the intensity is calculated as the sum of intensity on the localized nanowires (single-nanowire analysis, see Methods and Supporting Information Section 4.2). Left and right are the same image with and without the detections highlighted. (c,d) Epitaxy GaP nanowire platforms were evaluated in the same way as the aerotaxy nanowire platforms. (e,f) On planar glass slides, the intensity is extracted as the sum of intensity per pixel for all the ROIs (bulk analysis, see Methods and Supporting Information Section 4.2). The scale in all images is 0.27 μm/pixel as shown at the bottom right corner. The apparent difference on size between aerotaxy and epitaxy lit nanowires is considered in the analysis in the Section 4 of the Supporting Information).

For nanowire platforms, we used single-nanowire analysis. Specifically, bright spots were precisely localized (Figure 3a–d) (Supporting Information Section 4.1). The exact position of each nanowire was determined using a least squares Gaussian algorithm,^66^ and the average pixel intensity per nanowire was calculated, after which the dark current was subtracted.^67,68^ The overall intensity was calculated as the sum of the intensity values from all individual nanowires in an ROI (*I*_S_). At least 10 ROIs per sample and analyte concentration were averaged.

The same analysis as for nanowires cannot be applied to planar surfaces, since there are no detectable spatially localized emitters (Supporting Information Section 5). Therefore, images of planar glass surfaces were analyzed as a whole (planar analysis), again using ImageJ-Fiji. Specifically, the sum of intensity values from all pixels (Figure 3e,f) in an ROI was calculated and averaged for at least 10 ROIs (*I*_S_) per sample and analyte concentration, removing the dark current contribution.

To obtain comparable results between single-nanowire analysis and planar analysis, and to consider the particularities of individual platforms, each surface was normalized (*I*_N_) to its own blank measurement (*I*_B_). For these blanks, the same surfaces and imaging were used in the absence of StvA647 (no fluorophore). In cases where no signal from single nanowires could be observed for blank and low concentrations, planar analysis was performed (see Supporting Information Section 4.2 for details on these calculations).

## 3 Results and Discussion

From the fluorescence images acquired after performing the bBSA/StvA647 assay, we determined the sum of signal intensity *I*_S_ and normalized to the corresponding blank (*I*_B_) for each type of surface, to obtain *I*_N_ = *I*_S_/*I*_B_ as a function of StvA647 concentration.

**Figure 4:**
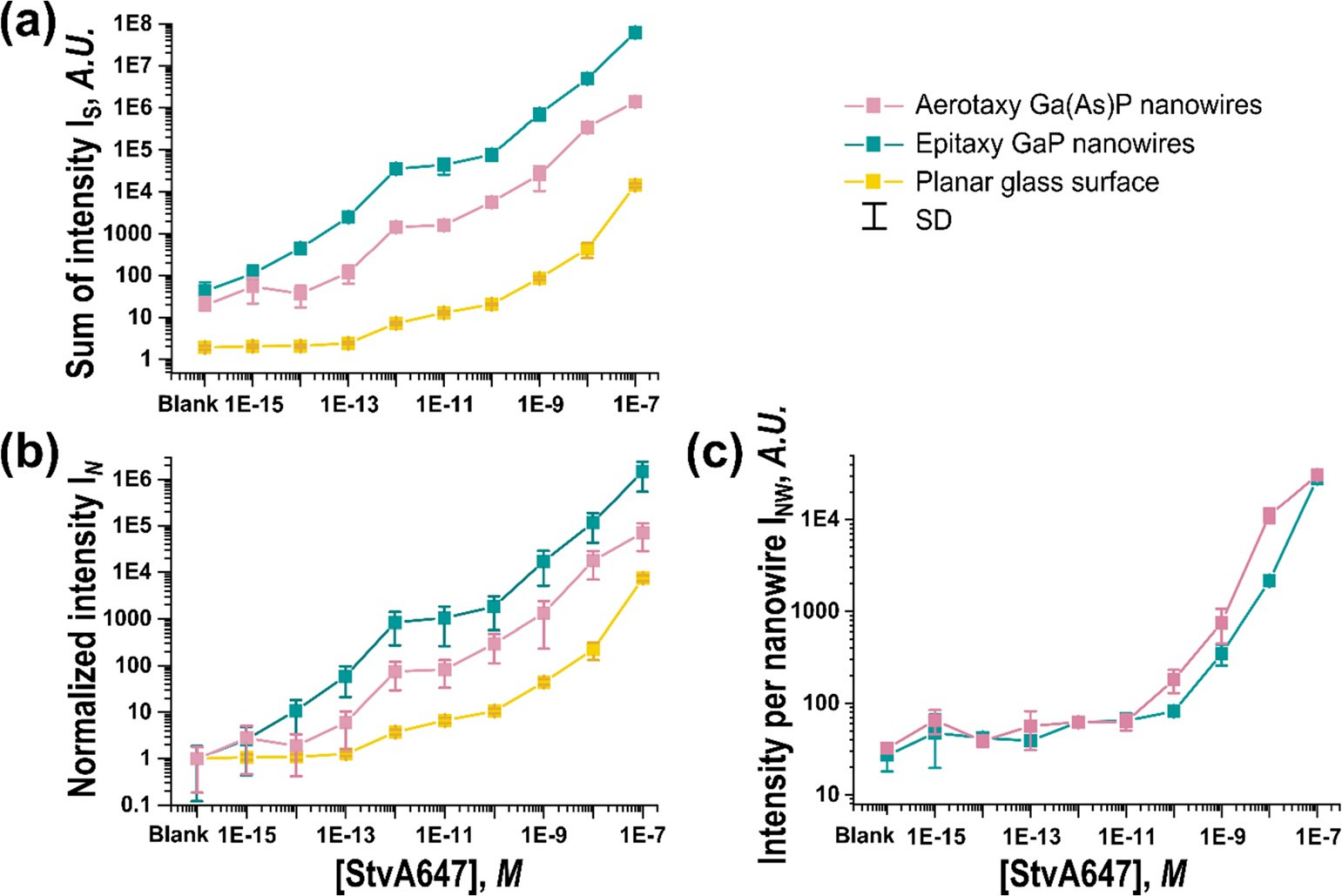
(a) Sum of signal intensity *I*_S_ as a function of concentration of StvA647 for aerotaxy nanowires, epitaxy nanowires, and planar controls, as indicated in the legend. (b) *I*_N_, the signal intensity of the samples normalized to the blank. (c) Average intensity per nanowire, for all detected bright nanowires *I*_NW_. In (b), due to the normalization, the value for the blank is always equal to one for the three surfaces. The error bars correspond to the standard deviation as an indication of the dispersion of the measurements of 10 different analyzed ROIs at the same surface and concentration (see Supporting Information Section 4 for details on how error bars are calculated). Note that both the vertical and horizontal axes are displayed on a logarithmic scale.

For both nanowire platforms, *I*_S_ and *I*_N_ were higher than for the planar glass control (Figure 4a,b). Specifically, compared to the planar glass surface, there was a ∼20-fold increase in *I*_N_ for aerotaxy nanowires and a ∼200-fold increase for epitaxy nanowires, along the range of evaluated concentrations. These results were reproduced in a second, similar experiment (Supporting Information Section 6).

Comparing aerotaxy nanowires to epitaxy nanowires, it is important to consider that in our experiments, the epitaxy platforms had ∼50 times higher density of nanowires contributing to the total *I*_S_ and *I*_N_. Crucially, when averaging the intensity per nanowire of all individual bright nanowires (*I*_NW_, Supporting Information Section 4.3), we find that individual aerotaxy and epitaxy nanowires displayed similar *I*_NW_ for low and medium StvA647 concentrations (1 fM to 10 pM) (Figure 4c). At higher StvA647 concentrations (0.1 to 100 nM), aerotaxy platforms performed better (by a factor of 2–10).

The key result of this study is that aerotaxy Ga(As)P nanowires, when individually analyzed, provide a similar signal intensity for optical biosensing as epitaxial GaP nanowires. At first, this may seem surprising, given that the aerotaxy nanowires show a higher degree of tapering and higher morphological variance than the epitaxy nanowires, and that many aerotaxy nanowires tilt away from the ideal vertical orientation. We therefore performed electromagnetic optics modeling of the response of the nanowires (Supporting Information Section 7). The modeling shows that the tapering of the aerotaxy nanowires is not expected to have a considerable negative effect for the optical response even at the tapering factor of ∼0.2 of aerotaxy nanowires used in this study, and thus, any minor deviations from ideal diameter and surface roughness are not expected to have a major impact on the waveguiding. However, an orientation with the wide end toward the substrate is expected to give higher excitation enhancement (Supporting Information Figures S8 and S9a,b). Similarly, modeling suggests that the observed tilt on our aerotaxy nanowires (0–15°) should not have a significant detrimental effect on nanowire performance for fluorescence detection (Supporting Information Figure S10).

An additional difference between the two nanowire platforms is the difference in the thickness *t*_c_ of the SiO_2_ layer. Specifically, as a result of the fabrication process used for the aerotaxy nanowires, they had *t*_c_ = 30 nm. These were benchmarked against the epitaxy nanowires for which we used *t*_c_ = 10 nm which is optimal for their performance. Indeed, optics modeling shows that a decrease of *t*_c_ from 30 to 10 nm for this type of nanowires might actually increase the signal by approximately a factor of ∼2 (Supporting Information Figure S9), predicting a higher excitation enhancement for the epitaxy nanowires. However, this effect might be partially counteracted by the fact that the total surface area per aerotaxy nanowire is about 15% larger than that of epitaxy nanowires (see Table 1) such that, in particular at high analyte concentrations, a higher number of molecules can be expected to bind and thus, resulting in a correspondingly larger signal per individual aerotaxy nanowire. The effect of spacing variations of nanowires has also been modeled (Supporting Information Section 7) and the possible role of additional differences between aerotaxy and epitaxy nanowires, deriving from the distinct production processes, are discussed in Supporting Information Section 8, and are not expected to have a major influence on our results.

**Figure 5:**
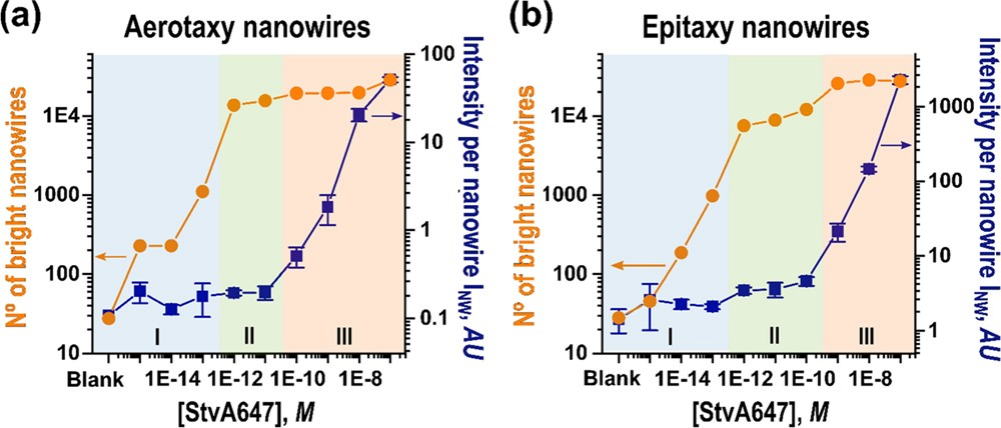
Comparison of the intensity per bright nanowire (blue squares), and the number of observed bright nanowires (orange dots), as a function of StvA647 concentration for (a) aerotaxy and (b) epitaxy. Three different concentration regimes (I–III) can be distinguished (see main text for discussion). Note that vertical and horizontal axes are displayed on a logarithmic scale.

Intriguingly, we find indications that both aerotaxy and epitaxy nanowires light up already upon single-molecule binding, consistent with earlier results.^27^ This becomes apparent when correlating the number of bright nanowires with the brightness of each nanowire as function of analyte concentration (Figure 5). For both types of nanowires, we can distinguish three concentration regimes, which we assign as follows: (I) At the lowest analyte concentrations, the intensity per nanowire is more or less constant, while the number of bright nanowires increases. This behavior is consistent with individual nanowires lighting upon binding of single StvA647 molecules, each labeled with ∼3 fluorophores. (II) At intermediate concentrations, we observe saturation of the number of available nanowires, but still with an only slightly higher average intensity per nanowire, consistent with only one, and rarely more than two, molecules per nanowire. (III) Finally, at the highest concentrations, in which all nanowires are bright, we observe gradual increase in brightness of each nanowire as multiple molecules bind to each nanowire. It is worth noting that for aerotaxy nanowires, the onset of Regime III starts at about one order of magnitude lower concentrations of StvA647 compared to epitaxy nanowires. This is consistent with the about 50 times lower density of aerotaxy nanowires, which suggests that the number of bright nanowires saturate at a lower analyte concentration than for epitaxy nanowires.

## 4 Conclusions

In conclusion, we find, using a biotin/streptavidin-fluorophore test assay, that vertical aerotaxy Ga(As)P nanowires offer an equivalent signal enhancement performance per nanowire (*I*_NW_) for optical biosensing as high-quality, epitaxially grown GaP nanowires. We find indications that both nanowire systems are capable of single-molecule detection using a standard epifluorescence microscope for imaging. A precise determination of the achievable LOD is out of the scope of this paper and would also depend on the specific capture and analyte system used for a given biosensing application. However, given the measured *I*_S_ and *I*_N_ for aerotaxy nanowires, compared to the planar glass control, it is reasonable to conclude that a signal discernible from the blank could prospectively be measured at concentrations at least 10 times lower than when using planar surfaces. Therefore, aerotaxy nanowires are a clear candidate for improving the detection limit of low-abundance molecules, when the expected signal is usually low. Because aerotaxy nanowires can be produced comparatively very cheaply in large amounts, this opens a pathway to mass-produced, low-cost surfaces for high-sensitivity optical biosensing, in a wide variety of biomedical diagnostic applications, including for point-of-care diagnostics. To reach this aim, additional optimization and characterization of aerotaxy nanowire platforms is required, with an emphasis on increasing the density and regularity of nanowires and modifying those parameters which could affect the homogeneity of measurements.

## Supporting Information Available

Further information on substrate preparation and nanowire growth, including SEM and TEM–EDS characterization, description of the flow chamber device, details on the surface properties of the evaluated platforms including fluorescence profiles, description of the numerical calculations required for extracting the signal intensity, data plots from additional experiments, preliminary modeling and simulations of the optical response of nanowires, and a brief discussion on the role of the composition and functionalization of the nanowire platforms on signal enhancement (PDF)

## Abbreviations

LOD: limit of detection
GaP: gallium phosphide
Ga(As)P: gallium (arsenide) phosphide
ZnO: zinc oxide
ELISA: enzyme linked immunoassay
InAs: indium arsenide
GaAs: gallium arsenide
MOVPE: metal–organic vapor phase epitaxy
SiO_2_: silicon dioxide
*I*_S_: sum of intensity of a sample with analyte
*I*_N_: normalized intensity
bBSA: biotinylated bovine serum albumin
StvA647: streptavidin-Alexa Fluor 647
*t*_c_: thickness of the oxide coating
*d*_T_: diameter at the thinner end of the nanowire
*d*_W_: diameter at the wider end of the nanowire
Au: gold
TMGa: trimethylgallium (Ga(CH_3_)_3_)
PH_3_: phosphine
DTL: displacement Talbot lithography
SiN: silicon nitride
ALD: atomic layer deposition
*L*: nanowire length
SEM: scanning electron microscopy
TEM−EDS: transmission electron microscopy−energy-dispersive spectroscopy
PBS: phosphate-buffered saline
RT: room temperature
EMCCD: electron-multiplying charge-coupled device
NA: numerical aperture
ROI: region of interest
*I*_B_: sum of intensity of the blank
AU: arbitrary units
*I*_NW_: average intensity per nanowire for all individual bright nanowires in a sample
