# Supplementary material for: Enhanced Optical Biosensing by Aerotaxy Ga(As)P Nanowire Platforms Suitable for Scalable Production
Source: ACS Appl Nano Mater. 2022 Jul 1;5(7):9063–71. doi: 10.1021/acsanm.2c01372 (PMC9315950; doi:10.1021/acsanm.2c01372)
Supplement: Supplementary file 1 — an2c01372_si_001.pdf [file an2c01372_si_001.pdf]

Supporting Information for: Enhanced Optical Biosensing by Aerotaxy Ga(As)P Nanowire Platforms Suitable for Scalable Production

Julia Valderas-Gutiérrez,[1,2] Rubina Davtyan,[1,2] Sudhakar Sivakumar,[1,2,†] Nicklas Anttu,[3] Yuyu Li,[4] Patrick Flatt,[4] Jae Yen Shin[1, 2,††] Christelle N. Prinz,[1,2] Fredrik Höök,[1,5] Thoas Fioretos,[6] Martin H. Magnusson[1,2] and Heiner Linke.[,1,2]*

[1]NanoLund, Lund University, P.O. Box 118, SE-22100 Lund, Sweden

[2]Division of Solid State Physics, Lund University, P.O. Box 118, SE-22100 Lund, Sweden

[3]Physics, Faculty of Science and Engineering, Åbo Akademi University, Henrikinkatu 2, FI-20500 Turku, Finland

[4]AlignedBio AB, Medicon Village, Scheeletorget 1, SE-22363 Lund, Sweden

[5]Department of Physics, Chalmers University of Technology, SE-41296 Göteborg, Sweden.

[6]Division of Clinical Genetics, Lund University, SE-22185 Lund, Sweden.

Corresponding Author

*E-mail: heiner.linke@ftf.lth.se.

Table of Contents

Section 1. Nanowire growth. 3

Section 1.1 Aerotaxy Ga(As)P nanowire growth. 3

Section 1.2 Epitaxy GaP nanowire growth. 4

Section 2. Further characterization of aerotaxy Ga(As)P nanowires. 6

Section 2.1. Additional SEM characterization. 6

Section 2.2. TEM-EDS characterization. 7

Section 3. Description of the microfluidic device. 9

Section 4. Detailed calculations for the extraction of signal intensity. 9

Section 4.1. Localization of bright nanowires using a gradient net threshold. 9

Section 4.2. Calculation of the total (I_S) and normalized (I_N) signal intensity. 10

Section 4.3. Calculation of the average intensity per nanowire (I_{NW}). 12

Section 5. Fluorescence profile on the evaluated surfaces. 12

Section 6. Additional experimental data. 13

Section 7. Optics modeling of aerotaxy Ga(As)P nanowires. 15

Section 8. Role of the composition and functionalization of nanowire platforms on signal enhancement properties. 22

Section 1. Nanowire growth.

This section describes details on each nanowire growth process used in this work.

Section 1.1 Aerotaxy Ga(As)P nanowire growth.

In aerotaxy, a custom-made reactor was required for the growth of Ga(As)P nanowires, catalyzed by Au nanoparticles in absence of a substrate. First, gold agglomerates were formed by an evaporation-condensation step in a high-temperature furnace and size-sorted (50 ± 15 nm), for a fine-tuned control on the optical properties of the nanowires, by a differential mobility analyzer (DMA), in combination with a particle charger (Figure S1a-b). Nanowire growth was initialized by mixing the seed particles with the Ga and P precursors. In particular, trimethylgallium (TMGa or $Ga(CH_3)_3$) was introduced on the reactor, at 430°C and under atmospheric pressure, to decompose and form an Au-Ga alloy which was further carried into a phosphine (PH_3) atmosphere (550°C). P atoms started to dissolve, leading to supersaturation and nucleation events, promoted by the continuous flow of precursors. This favored one-dimensional growth, guided by the seed particle and the formation of crystalline wires. While cooling, the nanowires exited the process, carried by an aerosol to be filtered and collected (Figure S1c-f).[1–3] The presence of residual As on

the walls of the reactor, from previous growth runs, gave rise to the non-zero As content in the Ga(As)P nanowires in this study (memory effect).[4]

After growth, aerotaxy Ga(As)P nanowires were coated with ~30 nm of SiO_2 (which is thicker than the ~10 nm for epitaxy nanowires described below, due to differences in the coating process) and aligned and embedded into an uncoated polymeric substrate by a proprietary technology (Figure S1g-h).[2]

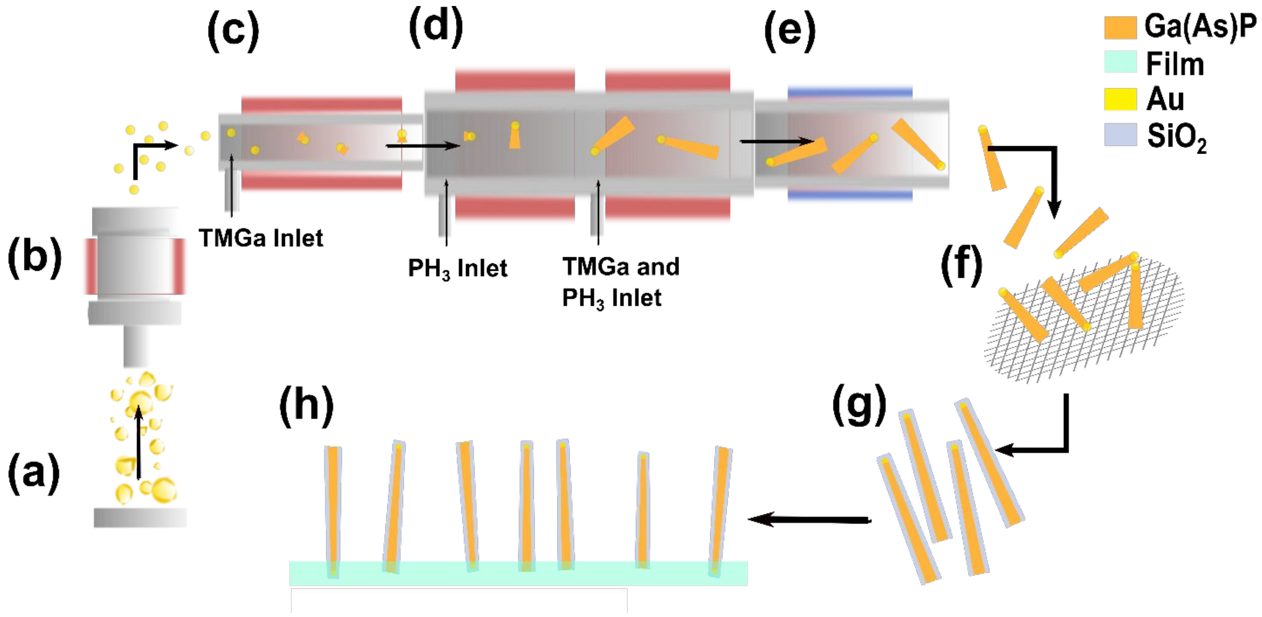

Figure S1. Schematic of the aerotaxy nanowire growth process, which starts with the Au agglomeration **(a)** and size-sorting in the DMA for a final compaction into spherical nanoparticles **(b).** The introduction of TMGa initiates the formation of a Ga-Au alloy **(c)**, and after the interaction with a phosphine atmosphere **(d)**, nucleation and supersaturation events lead to nanowire growth.

After a cooling-down step **(e)**, nanowires exit the reactor to be collected and filtered **(f).** Finally, they are coated with an oxide layer **(g)** and aligned in the polymeric substrate **(h)**.

Section 1.2 Epitaxy GaP nanowire growth.

Clean GaP(111)B wafers were processed to become the substrate used for nanowire growth. First, a ~70 nm SiN mask was deposited by plasma-enhanced chemical vapor deposition (PECVD), and then covered by a double layer of developable resists: SF 3S, a bottom anti-reflection coating (BARC); and PAR1085S90, a deep-ultraviolet resist (DUVR), which were used for pattern transfer. The nanopattern was initially transferred into the resists by displacement talbot lithography (DTL) from which it was transferred to the SiN mask by reactive ion etching (RIE). Finally, the Au seed particles were defined on the wafer by a standard evaporation method, depositing 60 nm of 24K gold, and a lift-off procedure was used to remove the resist, including the remaining gold on top.[5] An average density of 1.19 μm^{-2} was achieved (Figure S2a-f).

When the substrate was ready, GaP nanowires were grown by metal-organic vapor phase epitaxy (MOVPE) in a vapor-liquid-solid (VLS) mode, using an appropriate reactor (Aixtron 200/4). Succinctly, TMGa, phosphine and hydrogen chloride (HCl) were introduced into the reactor at 480°C. TMGa and phosphine dissolved in the Au particles, forming a liquid alloy which led to

supersaturation, and the nucleation of the crystal phase was initiated. The growth of the nanowires was maintained by the continuous supply of the precursors, controlling growth times, temperature and precursor ratios to achieve the desired diameter and length. Finally, the grown nanowires on the substrate were coated with a 10 nm-layer of SiO_2 by atomic layer deposition (ALD) and characterized afterwards by scanning electron microscopy (SEM). (Figure S2g-h).[3,5,6]

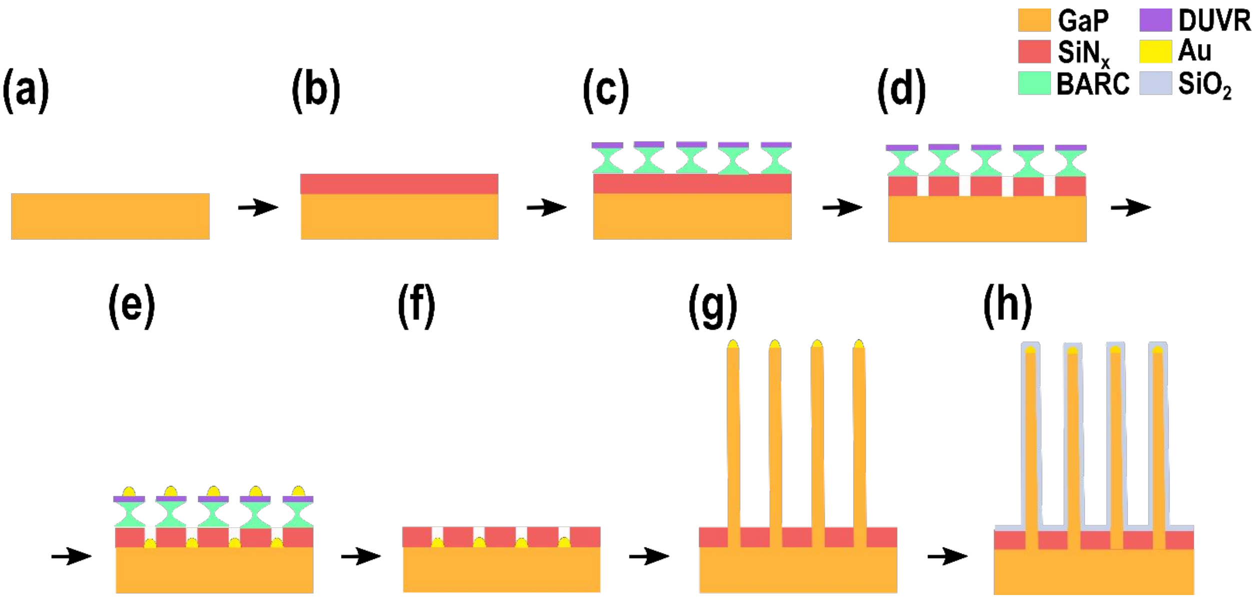

Figure S2. Schematic representation of the wafer processing and nanowire growth for epitaxy GaP nanowires. On a clean GaP(111)B wafer **(a)**, a SiN mask is deposited by PECVD **(b)**, and after the double-resist coating (BARC + DUVR), the pattern is transferred first to the resists by DTL **(c)** and then to the SiN mask by RIE **(d)**. Later, the Au seed particles were deposited by evaporation **(e)** and the excess gold and remaining resist layers were removed by a lift-off method **(f)**. GaP

nanowires are grown by MOVPE, catalyzed by the Au particles, to achieve the desired dimensions **(g)**, and coated with 10 nm of SiO_2 by ALD **(h)**.

Section 2. Further characterization of aerotaxy Ga(As)P nanowires.

Section 2.1. Additional SEM characterization.

Additional images were acquired in a SEM - Hitachi SU8010 using a voltage of ~15 KV for further characterization of the aerotaxy nanowires, including a close detail on the SiO_2 coating

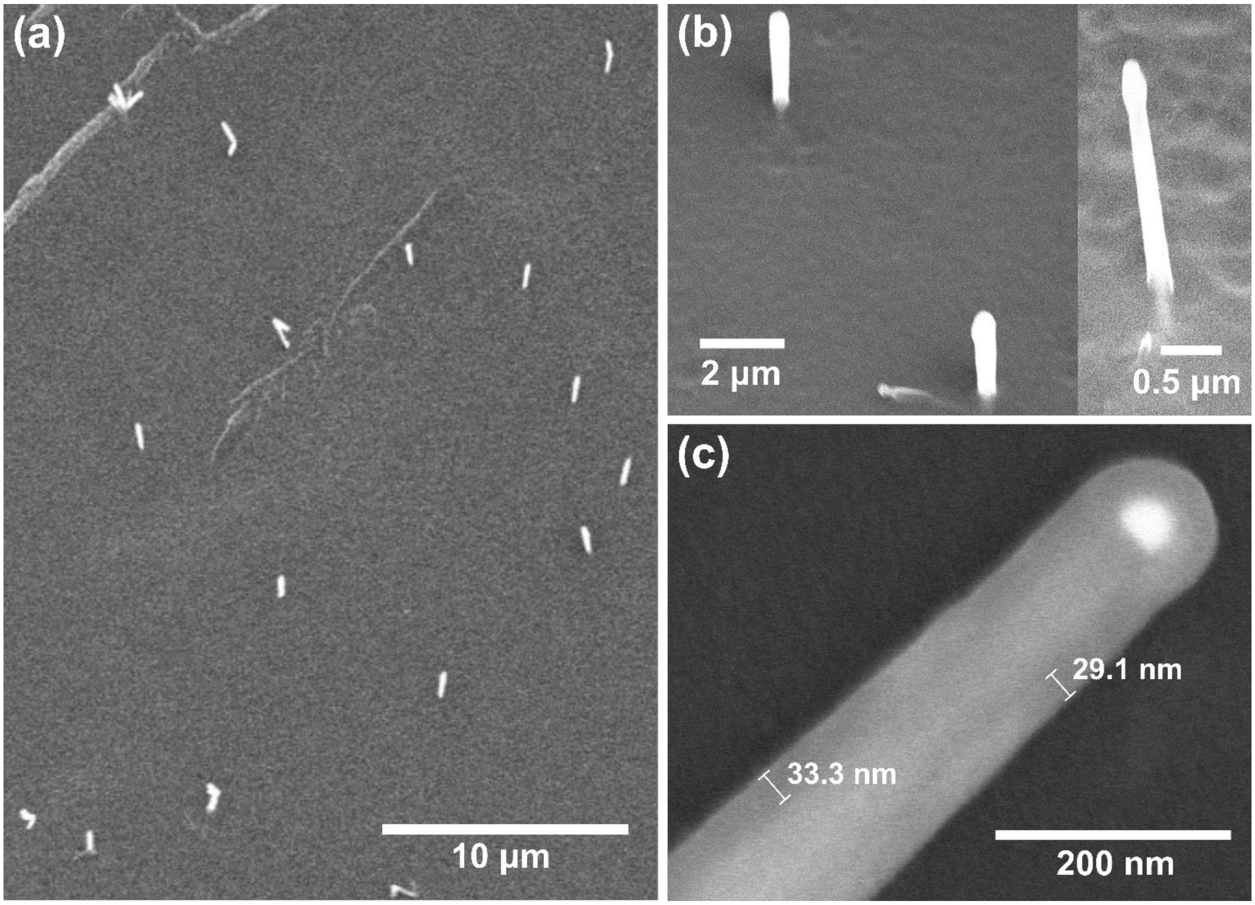

(Figure S3).

Figure S3. SEM images of aerotaxy Ga(As)P nanowires showing **(a)** a broad area with vertically aligned nanowires in a polymer film (stage tilt 30°) and **(b)** close-up view of individual nanowires including **(c)** a detail of the SiO_2 layer coating a nanowire, with a thickness of $t_c = 30$ nm.

Section 2.2. TEM-EDS characterization.

TEM-EDS measurements were performed on an additional batch of aerotaxy nanowires grown under the same growth conditions as the ones involved in this work. The TEM measurements were performed with a Hitachi 3300FS FETEM, in STEM mode. These wires exist in pure zincblende structure with twin plane defects, as corroborated in previous characterizations of similar samples (Figure S4).[2]

The EDS analysis showed that the wires are composed of Ga(As)P with ~13% of As in them (Table S1).

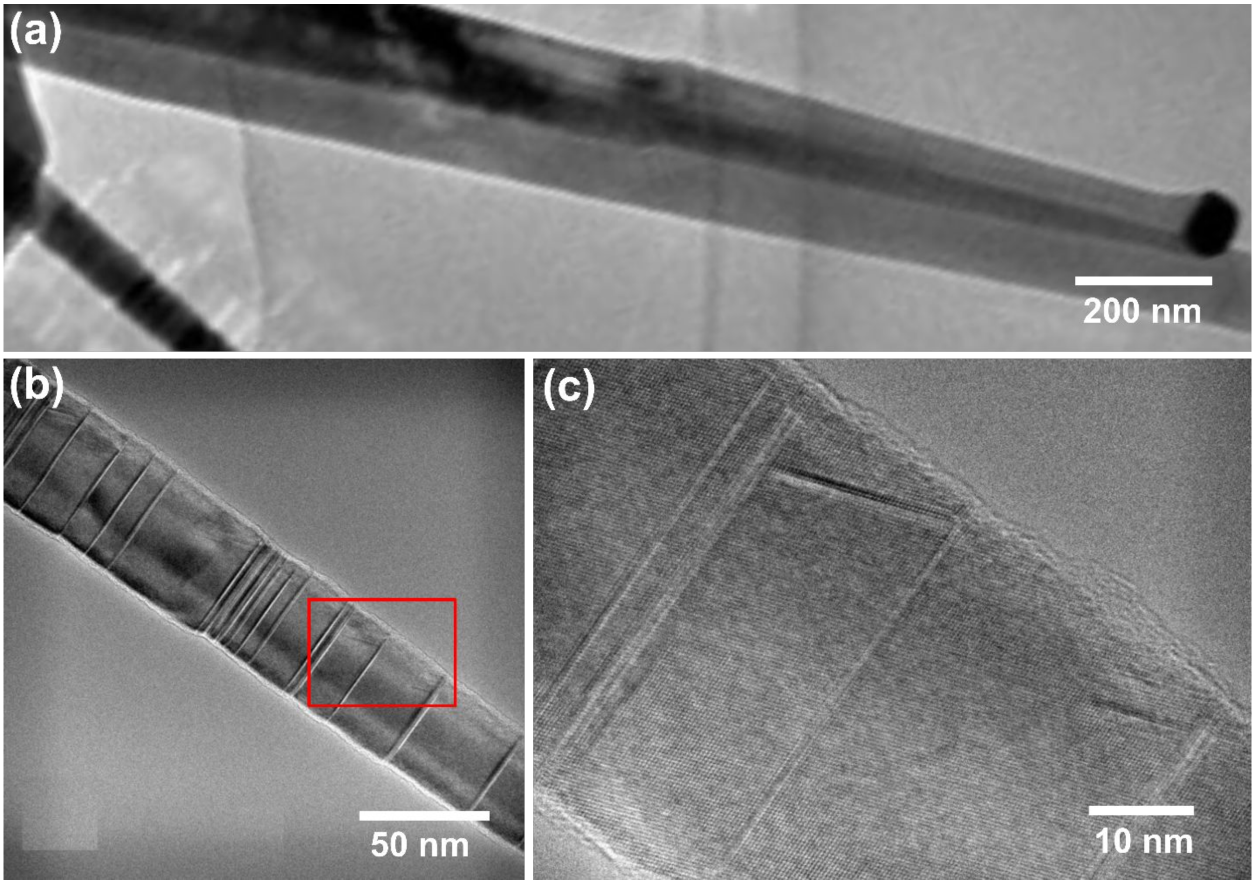

Figure S4. **(a)** TEM image of aerotaxy Ga(As)P nanowires grown under the same conditions as the ones involved in this work. In **(b)** and its higher magnification image **(c)**, corresponding to the red square in **(b)**, it is possible to appreciate the expected zincblende structure as described in previous characterizations.[2]

Table S1. EDS characterization of the aerotaxy Ga(As)P nanowires.

Spectrum Label	At %
Ga	40.3 ± 1.9
As	12.8 ± 2.5
P	46.9 ± 4.0
Au	0.0 ± 0.0
Total	100.0

EDS elemental quantification for the aerotaxy nanowires, showing the non-zero content of As, due to the memory effect of the reactor.

Section 3. Description of the microfluidic device.

For the fluorescence-based bioassays performed in this work, we used in all cases 6-channel flow chambers from ibidi, Germany (sticky-Slide VI 0.4).[7] This is a bottomless 6-channel slide that can be sealed by a glass coverslip (the SiO_2-coated planar glass reference in our assay) thanks

to the self-adhesive bottom. Each channel (17×3.8×0.4 mm) has a volume of 30 µl plus the two reservoirs with 60 µl each (Figure S5).

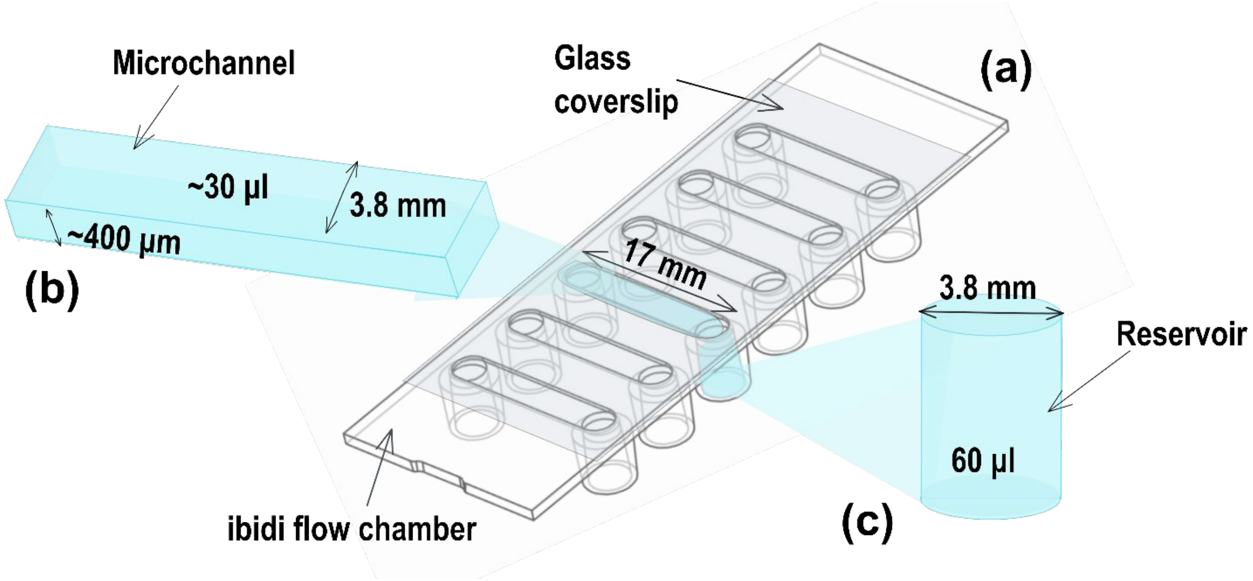

Figure S5. Schematic representation of a 6-channel ibidi flow chamber used in this work **(a)**, in which the volumes and the dimensions of the microchannels **(b)** and the reservoirs **(c)** are included. The drawing of the ibidi sticky-slide **(a)** was reproduced from the website of the product.[7]

Section 4. Detailed calculations for the extraction of signal intensity.

Section 4.1. Localization of bright nanowires using a gradient net threshold.

To localize single nanowires, local maxima are detected in the image by identifying the brightest pixels by performing image dilation.[8] For each local maximum, the net gradient (G) is calculated according to a user defined box size, as described elsewhere:[9]

$$G = \sum g_i \overrightarrow{u_i}$$

where $g_i$is the central difference gradient at i^{th} pixel and $\overrightarrow{u_i}$ is the unit vector pointing to the center of the box. Due to central symmetry of the point spread function corresponding to a single nanowire, true detections will have larger net gradient, hence setting an appropriate gradient threshold will significantly decrease the number of false positives obtained via image dilation.

Section 4.2. Calculation of the total (I_S) and normalized (I_N) signal intensity.

To obtain the sum of intensity I_S per each StvA647 concentration, the average pixel intensity of each identified nanowire $\bar{I}_i$ is calculated:

$$\bar{I}_i = \frac{\sum_{k=1}^{M}(I_{i,k} - I_{DC})}{M} \quad (1)$$

For each $\bar{I}_i$ of a localized nanowire, $I_{i,k}$ is the pixel intensity, M is the number of pixels per localized nanowire and I_{DC} is the intensity of the dark current, which was previously measured experimentally and is equal to 500. M (Equation 2) is defined according to the standard deviation estimated from fitted Gaussian point-spread function (PSF):

$$M = (2\sigma_x + 1) \times (2\sigma_y + 1) \quad (2)$$

where σ_x and σ_y are respectively the Gaussian PSF standard deviations in pixels in x and y directions, normally yielding $M = 9$.

If N bright nanowires were detected in a region-of-interest (ROI), the overall fluorescence signal I_{tot} can be calculated as the sum of the intensities of all detected nanowires (Equation 3). Finally, we get I_s as the average value for the number of ROIs (R = 10) at the same platform and concentration of StvA467 (Equation 4):

$$I_{tot} = \sum_{i=1}^{N} \bar{I}_i \quad (3)$$

$$I_S = \frac{\sum_R I_{tot}}{R} \quad (4)$$

For the calculation for I_S at those images of the blank (I_B in that case) and samples at low concentrations of StvA647, where no well-localized emitters were detected, the planar analysis approach is used, in the same way as for flat surfaces. Here $\bar{I}_i$ is the average intensity per pixel (Equation 5), $I_{i,k}$ is the intensity of an individual pixel, I_{DC} is the intensity of the dark current (I_{DC}= 500) and P is the total number of pixels of a ROI (200x200). Then, I_S is obtained with the same procedure as for nanowire platforms (Equations 6-7).

$$\bar{I}_i = \frac{\sum_{k=1}^{P} (I_{i,k} - I_{DC})}{P} \quad (5)$$

$$I_{tot} = \sum_{i=1}^{N} \bar{I}_i \ (6)$$

$$I_S = \frac{\sum_R I_{tot}}{R} \ (7)$$

Finally, to calculate I_N, the I_S of the samples with StvA647 is normalized to the I_B of the blank without StvA647 (Equation 8) and the error, corresponding to the standard deviation (SD), is propagated accordingly,[10] assuming that variables are uncorrelated (Equation 9). Since ROIs were manually selected, I_S is averaged over the ROIs and the SD shows ROI to ROI deviation:

$$\frac{I_S}{I_B} = I_N \ (8)$$

$$SD_N = \frac{I_S}{I_B} \cdot \sqrt{\left(\frac{SD_S}{I_S}\right)^2 + \left(\frac{SD_B}{I_B}\right)^2} \ (9)$$

Section 4.3. Calculation of the average intensity per nanowire (I_{NW}).

First, the intensity of each individual nanowire $\bar{I}_i$ is averaged to the number of bright nanowires N in a ROI (Equation 10):

$$I_{Ai} = \frac{\sum_{i=1}^{N} \bar{I}_i}{N} (10)$$

Then, I_{Ai} is averaged to the number of ROIs (R = 10) in a sample (Equation 11), to obtain the final values of the average intensity per bright nanowire (I_{NW}) which are represented in Figure 4c of the main text.

$$I_{NW} = \frac{\sum_R I_{Ai}}{R} \quad (11)$$

Section 5. Fluorescence profile on the evaluated surfaces.

As indicated in the main text, the extraction and comparison of the signal intensity values for the three evaluated surfaces is not trivial, given their differences. For both nanowire samples, we can appreciate well-localized emitters as peaks of intensity along the image, clearly identifiable from the background; on the other hand, the signal intensity of the glass slides is homogeneous along the measured area, thus no single-nanowire approach is acceptable, and ROIs are analyzed using planar analysis (Figure S6).

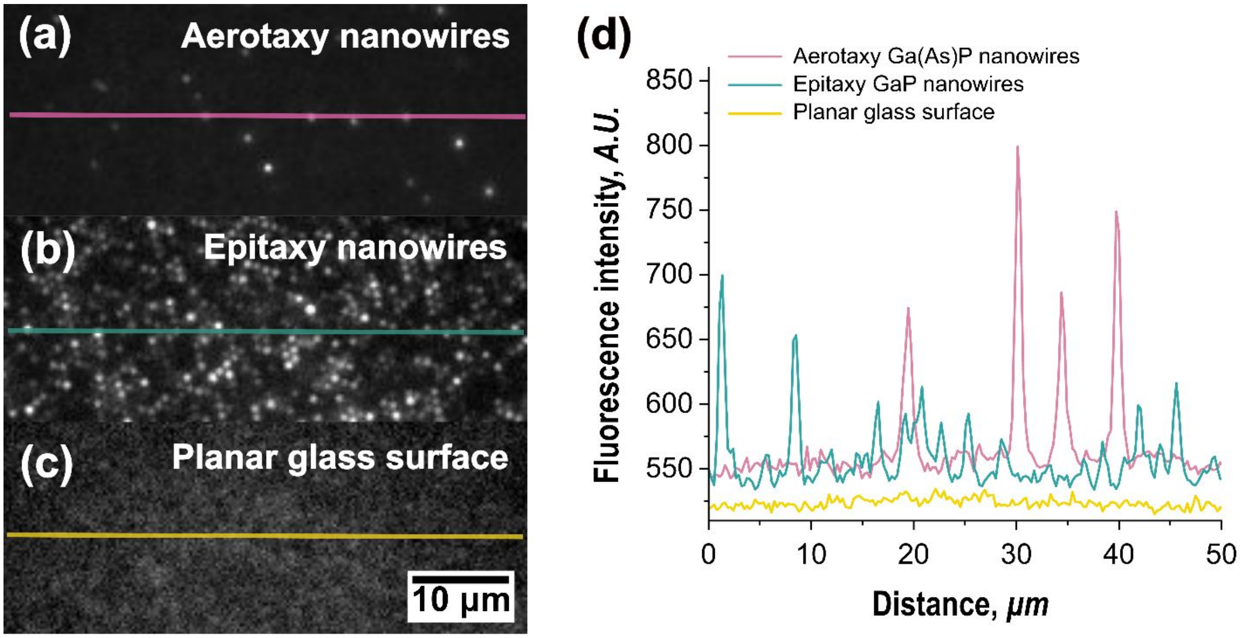

Figure S6. Fluorescence images of **(a)** aerotaxy Ga(As)P nanowires, **(b)** epitaxy GaP nanowires and **(c)** planar glass platforms, for a concentration of 100 pM of StvA647, in which the fluorescence profile **(d)** is represented as variations of signal intensity per pixel across each surface. We can observe the peaks of intensity for the lit nanowires, especially bright for aerotaxy, and more cluttered for denser epitaxy platforms; in contrast, the intensity from the planar glass doesn´t show substantial variations along the surface.

Section 6. Additional experimental data.

A second, similar experiment was performed following the same experimental design and analysis as in the main experiment described above and in the main text, although some of the conditions couldn't be entirely replicated: the quality of the epitaxy batches was poorer and the presence of some sort of contamination was suspected. Hence, the two experiments must be considered and evaluated separately. The details on the morphological characterization of the involved batches are shown in Table S2.

Even though the signal intensity was lower, the behavior when comparing the three platforms is consistent with the results in the main experiment and signal increased at least by a factor of 10

and 100 for aerotaxy and epitaxy respectively, when comparing to the glass, with regard to both I_S and I_N (Figure S7).

Table S2. Morphological characterization for aerotaxy and epitaxy nanowires in the additional experiment.

	Aerotaxy nanowires	Epitaxy nanowires
d_W (nm)	137 ± 21	110 ± 6
d_T (nm)	89 ± 21	105 ± 6
Average diameter (nm)	113 ± 15	108 ± 4
Length, L (μm)	2.23 ± 0.39	2.45 ± 0.09
t_c of SiO_2 (nm)	32 ± 6	11 ± 1
Spacing (μm)	≈ 5-10	0.99
Density (nanowires/μm^2)	0.016	0.790 (66% of 1.19, considering only the non-defective nanowires)
Tapering factor	0.217 ± 0.107	0.023 ± 0.016
Tilt away from vertical (degrees)	0 – 15	Negligible for non-kinked nanowires
Lateral surface area per nanowire (μm^2/nanowire)	1.25 ± 0.18	0.99 ± 0.04
Effective surface area (μm^2/μm^2)	0.020 ± 0.003	0.78 ± 0.04

d_W, d_T, L and t_c were measured using SEM images for N ≥ 30 nanowires. The average diameter was calculated as $(d_W + d_T)/2$. For aerotaxy films, L was measured on horizontal nanowires before being embedded into the polymer film. Spacing between nanowires in epitaxy samples was achieved by using a specific mask for DTL. Density values were determined from optical microscopy images for aerotaxy and SEM images for epitaxy (N ≥ 600). In the case of epitaxy platforms, we consider the density of non-kinked nanowires since they are the only ones whose signal can be detected. The tapering factor (N ≥ 30) is calculated as $(d_W - d_T)/(d_W + d_T)$. The lateral surface area per nanowire was calculated according to the formula for a truncated cone: $\frac{\pi}{2}(4t_c + d_W + d_T)\sqrt{\frac{1}{4}(d_W - d_T)^2 + L^2}$. The effective surface area is the surface area per nanowire multiplied by the density of nanowires.

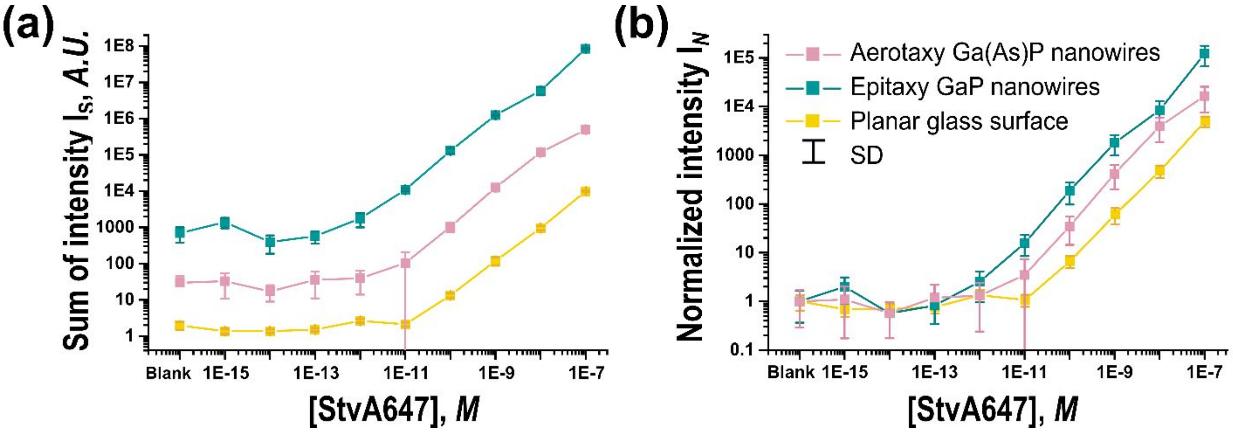

Figure S7. **(a)** Sum of signal intensity I_S as a function of concentration of StvA647 and **(b)** the signal intensity normalized to the blank I_N, for aerotaxy (pink), epitaxy (blue) and planar glass (yellow) platforms. A similar result as in the main experiment is observed, and the total signal enhancement of nanowire platforms is maintained when comparing with the glass platforms, and an improvement in I_N of one and two orders of magnitude is exhibited for aerotaxy and epitaxy platforms, respectively. Note that vertical and horizontal axes are displayed on a logarithmic scale.

Section 7. Optics modeling of aerotaxy Ga(As)P nanowires.

The optics modeling of the excitation enhancement for aerotaxy nanowires referred to on the main text is described in this section. For the modeling, two different possible orientations were considered (Figure S8), for aerotaxy nanowires of a diameter at the wider end d_W, a diameter at the thinner end d_T, length of L and a SiO_2 shell of thickness t_c. A fluorophore is placed on top of the shell and the excitation light is incident from a cone with half-angle defined by $\theta_{NA} = \text{asin}(NA/n_{top})$, where NA is the numerical aperture (NA = 1) and n_{top} is the refractive index for the liquid above the substrate (n_{top} = 1.33). The substrate (polymer film) is modeled with n_{sub} = 1.4. For the simulated $GaAs_xP_{1-x}$ with $x \approx 0.2$, a correspondingly weighted refractive index between that of GaP[11] and GaAs[12] at the simulated wavelength was used. Since the lowest energy band-to-band transition of $GaAs_xP_{1-x}$ with $x \approx 0.2$ is below 640 nm in wavelength,[13] only the real part of the refractive index of GaAs was considered in this weighting. This resulted in a refractive index of 3.4179 for the simulations. For the SiO_2 coating, a tabulated value[14] of 1.457 was used. Note that for simplicity, the Au particle was not included in the modeling.

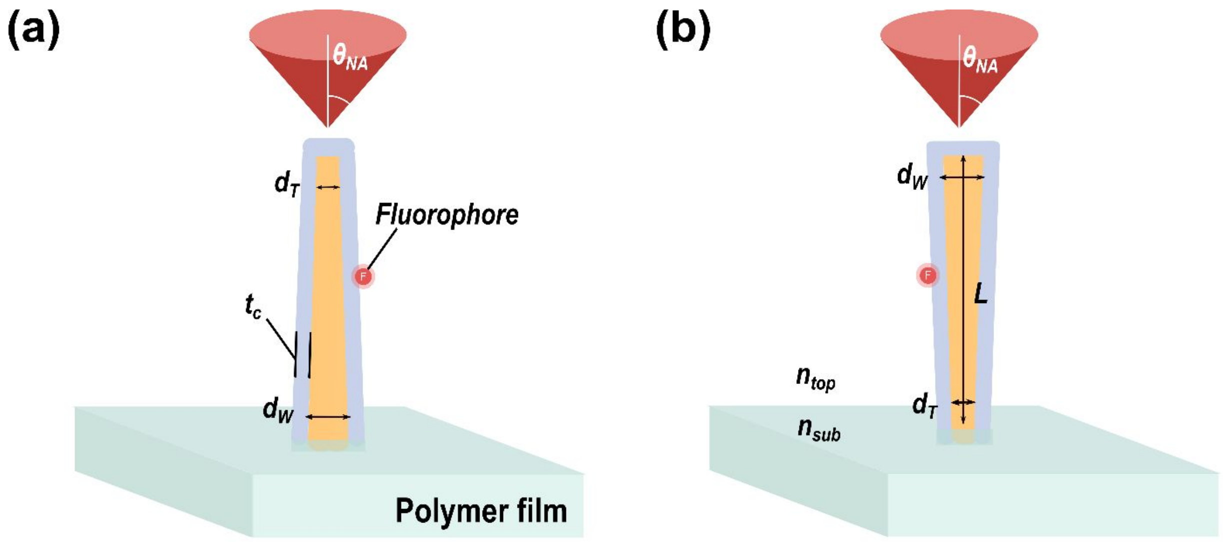

Figure S8. Schematics of the geometry for the optics modeling of a nanowire with wide and thin diameters d_W and d_T, respectively, length L and a SiO_2 shell with thickness t_c, with a fluorophore on top of it. Aerotaxy nanowires can display either an orientation of d_W towards substrate **(a)** or d_T towards substrate **(b)**. The incident light comes from a cone with half-angle of $\theta_{NA} = \text{asin}\,(NA/n_{top})$, where NA = 1, the refractive index for the liquid above the substrate is n_{top} = 1.33, and for the substrate, n_{sub} = 1.4.

The modeling for the excitation enhancement was performed by solving the Maxwell equations for the excitation wavelength of 640 nm with the finite-element method in Comsol Multiphysics, similarly as detailed elsewhere.[15] To model the excitation enhancement, we consider the

enhancement of the intensity of the light at $\mathbf{r}_{\text{fluor}}$, the position of the fluorophore. For each incidence angle and polarization state within the numerical aperture, we performed the modeling of an incident plane wave. This gives the enhancement $|\mathbf{E}(\mathbf{r}_{\text{fluor}})|^2/|\mathbf{E}_0|^2$ where $\mathbf{E}_0$ is the amplitude of the incident plane wave and $\mathbf{E}(\mathbf{r}_{\text{fluor}})$ is the resulting electric field at the position of the fluorophore, which differs from $\mathbf{E}_0$ due to the diffraction of light by the nanowire core, the oxide layer, and the polymer substrate. To obtain the excitation enhancement for light incident within the numerical aperture and to model the incoherent illumination in a widefield microscope, we averaged the above enhancement over the incidence angles within the numerical aperture and for both polarization states. In this averaging, we assumed equal incident intensity from any given solid angle within the numerical aperture. The normalization for the intensity of the incident light was chosen such that an enhancement of 1 in the results below corresponds to the excitation intensity on a fluorophore in a homogeneous liquid-surrounding of n_{top}. We ascertained that our choice for the discretization of the incidence angles in the averaging was fine enough to not affect the results.

The tapering factor was defined as $(d_W - d_T)/(d_W + d_T)$ and the average diameter as $(d_W + d_T)/2$, as in the experiments. The enhancement was averaged here over a 10-nm-thick volume directly on top of the oxide coating in the radial direction, as depicted in Figure S8 for one possible

position of the fluorophore. Thus, a higher weight was given in the averaging to axial positions, where the local diameter is larger in a tapered nanowire. The results are shown in Figure S9 for the two possible geometries depicted in Figure S5 and for nanowires with t_c = 30 and 10 nm.

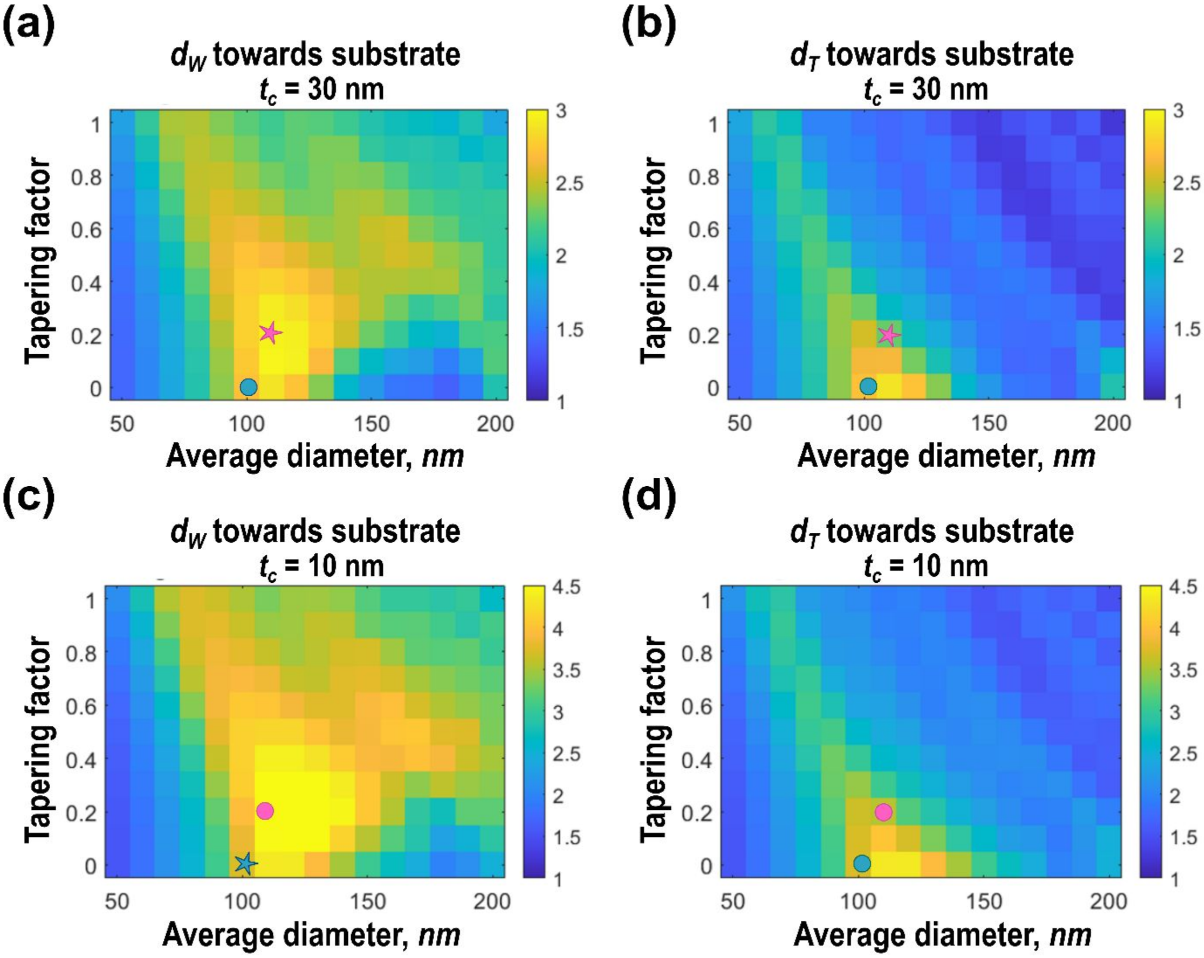

Figure S9. Excitation enhancement at the wavelength of 640 nm for aerotaxy nanowires with L = 2000 nm and light incident from NA = 1. The modeling was performed for both possible

orientations (d_W or d_T towards the substrate), with t_c = 30 nm **(a-b)** and t_c = 10 nm **(c-d).** The excitation enhancement is evaluated in terms of the tapering factor and the average diameter of the nanowire. The symbols indicate the aerotaxy (pink) and epitaxy (blue) nanowire dimensions relevant for this study, although only the stars represent real conditions involved in the experiments.

It is worth mentioning that the enhancement for an average diameter of 110 nm and tapering factor of 0.2 of our aerotaxy nanowires (pink symbols) shows an increase by a factor of ~1.5 when decreasing t_c = 30 nm to 10 nm. Since the fluorescence peak wavelength of 670 nm for the Alexa Fluor 647 dye is close to the modeled excitation wavelength of 640 nm, also the emission directionality is expected to exhibit a similar dependence due to the Lorentz's reciprocity,[16] therefore showing a prospective enhancement by a factor of approximately $1.5^2 \approx 2$ in the overall signal if reducing t_c from 30 nm to 10 nm. Moreover, the effect of the orientation of the nanowires on signal enhancement is not trivial, and when the wider end (d_W) is facing towards the substrate, we observe higher values for a broader range of average diameters and tapering factors (for the relevant dimensions of the aerotaxy nanowires in this study an improvement by a factor of ~1.2 is displayed), which might be part of the explanation for the higher variability on I_{NW} for aerotaxy

nanowires, translated into a higher SD, in comparison with epitaxy nanowires (Figure 4c of the main text).

We also simulated the effect from the tilting of the aerotaxy nanowires on the excitation enhancement. This was achieved by modeling the effect from tilting the incidence cone relative to the nanowire axis by θ_{tilt} (Figure S10a), which is not necessarily completely equal to the effect from tilting the nanowire axis relative to the substrate and incidence cone. However, we believe that these results give a good indication of the expected effect from tilting of the nanowire axis, which would be a numerically considerably more demanding modeling study since the tilting of the nanowire breaks the symmetry of the problem to a larger degree than the tilting of the incidence cone.

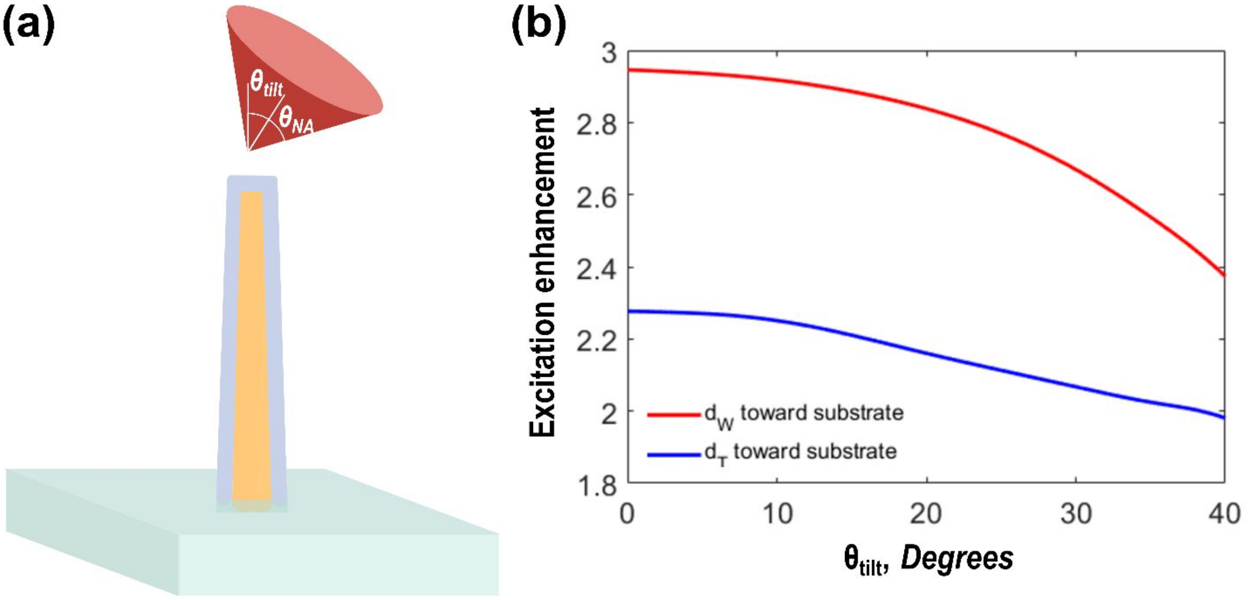

Figure S10. Schematic of the tilting of the incidence cone relative to the nanowire axis by θ_{tilt} (in contrast with Figure S9, where $\theta_{tilt} = 0$) **(a)** and modeled excitation enhancement **(b)** for varying θ_{tilt} and the two possible geometries from Figure S8. The results are for NA = 1 and tapered nanowires with $d_W = 132$ nm, $d_T = 88$ nm, $L = 2000$ nm and $t_c = 30$ nm, close to the experimental dimensions of the aerotaxy nanowires, as these d_W and d_T correspond to an average diameter of 110 nm with a tapering factor of 0.2.

As our results show, the effect from the tilting starts having a significant impact on the excitation enhancement at an angle above ~20 degrees (Figure S10b). This tilting of the incidence cone includes the directionality of the excitation, and by the Lorentz's reciprocity,[16] the directionality of the emission as well, thus a similar behavior with this tilt angle is expected also for emission collection efficiency due to the small offset between the fluorescence peak wavelength of 670 nm for the dye and the modeled excitation wavelength of 640 nm.

Since the measured tilt angle of the aerotaxy nanowires does not reach such high tilting degree (instead showing a range of tilt from 0 to 15°), the impact of the tilting shouldn't be as significant on the performance of aerotaxy nanowires as the thickness of the coating, the orientation of the nanowire or, to a smaller extent, the tapering is.

To conclude, we have elucidated the effect of neighboring nanowires, by modeling a square array of nanowires with period P (Figure S11). For $P < 1000\ nm$ (that is $\approx 1.5\lambda$), we notice a drop in the enhancement. At the smallest array period of 200 nm modeled, the enhancement is just 14% of that in the single nanowire. In contrast, when the nanowires are spaced by a distance of approximately 1.5λ or more, we expect an enhancement very close to that of the single nanowire. We have ascertained by varying the diameter, at small and intermediate values for the period, that the drop in enhancement is not due to a shift in the wavelength vs. diameter response with decreasing array pitch. Indeed, this drop in the enhancement with decreasing array period is in line with the decreased possibility to couple to the waveguide mode in each nanowire due to competition between neighboring nanowires, as seen also in absorption studies with nanowires.[17,18]

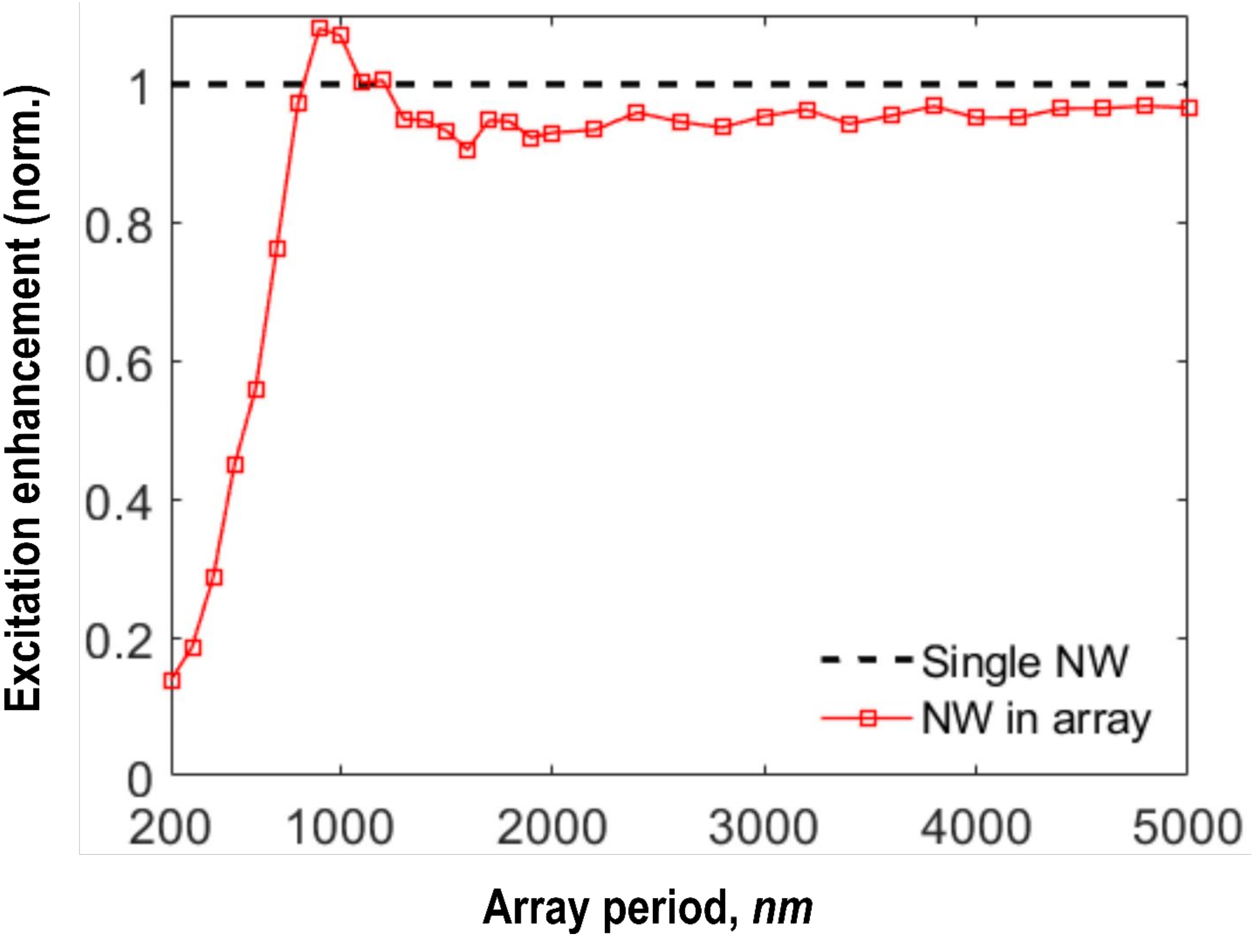

Figure S11. Simulated excitation enhancement at the wavelength of $\lambda = 640\,nm$ for aerotaxy nanowires with $L = 2000$ nm and $t_c = 30\,nm$. Here, we study possible effects from neighboring nanowires by placing the nanowires in a square array of period P. For simplicity, non-tapered nanowires of 110 nm in diameter were modelled. For convenience of numerical simulations, these simulations are performed in the limit of $NA \to 0$, that is, for normally incident light toward the

array. The results are normalized to the enhancement in the single nanowire, which is approximately a factor of 3 higher than at $NA = 1$ (described in a submitted manuscript).

Section 8. Role of the composition and functionalization of nanowire platforms on signal enhancement properties.

Because the aerotaxy and epitaxy nanowire platforms are fabricated along entirely different routes, there are a number of differences that may affect optical biosensing performance. In addition to those discussed in the main text, the following factors may also play a role:

(1) The epitaxial growth of the nanowires occurs in a GaP substrate, SiO_2-coated by ALD, together with the nanowires. In contrast, coated aerotaxy nanowires are embedded to an uncoated polymer film. This SiO_2 shell, among other properties, increases the hydrophilicity of the surface and hence, the binding of molecules like BSA.[19] Hence, both coated GaP nanowires and substrate would have a similar affinity for bBSA, potentially competing for the binding of fluorophores. However, for aerotaxy, the specific binding of BSA would be promoted mainly on the coated nanowires. This leads to differences in the available surface area for binding analyte.

(2) The presence of a remaining ~70 nm SiN mask on the GaP substrate in epitaxy nanowire platforms (Figure S2) could have an effect on the photonic effects underlying the lightguiding behavior for the epitaxy wires.

(3) The composition of aerotaxy nanowires (Ga(As)P, that is, including remnants of As) is different from the pure GaP epitaxy nanowires. However, we expect this effect to be minimal in terms of lightguiding performance, given the very similar refractive indexes of GaP ($n \approx 3.3$)[11] and GaAs ($\mathrm{Re}(n) \approx 3.8$).[12] Our control simulations have shown that the optimum diameter for signal enhancement shifts linearly with the refractive index of the nanowire material, as expected for waveguide modes in the nanowire.[20]

ABBREVIATIONS

Ga(As)P, gallium (arsenide) phosphide;

DMA, differential mobility analyzer;

TMGa, trimethylgallium ($Ga(CH_3)_3$),

PH_3, phosphine;

GaP, gallium phosphide;

SiN, silicon nitride;

PECVD, plasma-enhanced chemical vapor deposition;

BARC, bottom anti-reflection coating;

DUVR, deep-ultraviolet resist;

DTL, displacement Talbot lithography;

RIE, reactive ion etching;

Au, gold;

MOVPE, metal-organic vapor phase epitaxy;

VLS, vapor-liquid-solid;

HCl, hydrogen chloride;

SiO_2, silicon dioxide;

ALD, atomic layer deposition;

SEM, scanning electron microscopy;

G, net gradient;

I_S, sum of intensity of a sample with analyte;

$\bar{I}_i$, average intensity of each identified nanowire (or average intensity per pixel);

$I_{i,k}$, intensity per individual pixel;

M, number of pixels per localized nanowire;

I_{DC}, intensity of the dark current;

PSF, point-spread function;

ROI, region of interest;

σ, Gaussian PSF standard deviation;

I_{tot}, sum of intensities per nanowire in a ROI;

N, number of detected wires;

R, number of ROIs;

StvA647, streptavidin-AlexaFluor647;

I_B, sum of intensity of the blank;

P, total number of pixels in a ROI;

I_N, normalized intensity;

SD, error (standard deviation);

I_{Ai}, average intensity per individual bright nanowire in a single ROI;

I_{NW}, average intensity per nanowire for all individual bright nanowires in a sample;

bBSA, biotinylated-bovine serum albumin;

d_W, diameter at the wider end of the nanowire;

d_T, diameter at the thinner end of the nanowire;

d_W, diameter at the wider end of the nanowire;

L, nanowire length;

t_c, coating thickness;

θ_{NA}, half-angle of inclination for the incident light;

NA, numeric aperture;

n_{top}, refractive index for the liquid above the substrate;

n_{sus}; refractive index for the substrate;

$\mathbf{r}_{\mathrm{fluor}}$, position of the fluorophore;

$\mathbf{E}_0$, amplitude of the incident plane wave;

$\mathbf{E}(\mathbf{r}_{\mathrm{fluor}})$, resulting electric field at the position of the fluorophore;

GaAs, gallium arsenide;

θ_{tilt}, additional degree of inclination for the incident light to simulate tilt nanowires.

REFERENCES

(1) Heurlin, M.; Lindgren, D.; Deppert, K.; Samuelson, L.; Magnusson, M. H.; Ek, M. L.; Wallenberg, R. Continuous Gas-Phase Synthesis of Nanowires with Tunable Properties. *Nature* **2012**, *492* (7427), 90–94. https://doi.org/10.1038/nature11652.

(2) Sivakumar, S.; Persson, A. R.; Metaferia, W.; Heurlin, M.; Wallenberg, R.; Samuelson, L.; Deppert, K.; Johansson, J.; Magnusson, M. H. Aerotaxy: Gas-Phase Epitaxy of Quasi 1D Nanostructures. *Nanotechnology* **2021**, *32* (2), 10. https://doi.org/10.1088/1361-6528/abbc23.

(3) Sivakumar, S. Understanding and Optimization of III-V Nanowire Growth in Aerotaxy. Ph.D. Thesis, Lund University, Lund, Sweden, 2021.

(4) Welser, E.; Guter, W.; Wekkeli, A.; Dimroth, F. Memory Effect of Ge in III-V Semiconductors. *J. Cryst. Growth* **2008**, *310* (23), 4799–4802. https://doi.org/10.1016/j.jcrysgro.2008.08.037.

(5) Flatt, P. AlP Sacrificial Layer for GaP NW Growth. PhD Thesis., Lund University, Lund, Sweden, 2020.

(6) Verardo, D.; Agnarsson, B.; Zhdanov, V. P.; Höök, F.; Linke, H. Single-Molecule Detection with Lightguiding Nanowires: Determination of Protein Concentration and Diffusivity in Supported Lipid Bilayers. *Nano Lett.* **2019**, *19* (9), 6182–6191. https://doi.org/10.1021/acs.nanolett.9b02226.

(7) ibidi. *sticky-Slide VI 0.4 | Self-Adhesive Underside*. https://ibidi.com/sticky-slides/65-sticky-slide-vi-04.html (accessed 2022-05-20).

(8) Soille, P. *Morphological Image Analysis*; Springer Berlin Heidelberg: Berlin, Heidelberg, 2004. https://doi.org/10.1007/978-3-662-05088-0.

(9) Schnitzbauer, J.; Strauss, M. T.; Schlichthaerle, T.; Schueder, F.; Jungmann, R. Super-Resolution Microscopy with DNA-PAINT. *Nat. Protoc.* **2017**, *12* (6), 1198–1228. https://doi.org/10.1038/nprot.2017.024.

(10) Taylor, J. R. *An Introduction to Error Analysis: The Study of Uncertainties in Physical Measurements*, 2nd ed.; University Science Books, 1997.

(11) Borghesi, A.; Guizzetti, G. Gallium Phosphide (GaP). In *Handbook of Optical Constants of Solids*; Elsevier, 1985; Vol. 1, pp 445–464. https://doi.org/10.1016/B978-0-08-054721-3.50021-6.

(12) Palik, E. D. Gallium Arsenide (GaAs). In *Handbook of Optical Constants of Solids*; Elsevier, 1985; Vol. 1, pp 429–443. https://doi.org/10.1016/B978-0-08-054721-3.50020-4.

(13) Vurgaftman, I.; Meyer, J. R.; Ram-Mohan, L. R. Band Parameters for III–V Compound Semiconductors and Their Alloys. *J. Appl. Phys.* **2001**, *89* (11), 5815–5875. https://doi.org/10.1063/1.1368156.

(14) Malitson, I. H. Interspecimen Comparison of the Refractive Index of Fused Silica*,†. *J. Opt. Soc. Am.* **1965**, *55* (10), 1205. https://doi.org/10.1364/josa.55.001205.

(15) Anttu, N.; Mäntynen, H.; Sorokina, A.; Kivisaari, P.; Sadi, T.; Lipsanen, H. Geometry Tailoring of Emission from Semiconductor Nanowires and Nanocones. *Photonics* **2020**, *7* (2), 23. https://doi.org/10.3390/PHOTONICS7020023.

(16) Anttu, N.; Mäntynen, H.; Sorokina, A.; Turunen, J.; Sadi, T.; Lipsanen, H. Applied Electromagnetic Optics Simulations for Nanophotonics. *J. Appl. Phys.* **2021**, *129* (13).

https://doi.org/10.1063/5.0041275.

(17) Nowzari, A.; Heurlin, M.; Jain, V.; Storm, K.; Hosseinnia, A.; Anttu, N.; Borgström, M. T.; Pettersson, H.; Samuelson, L. A Comparative Study of Absorption in Vertically and Laterally Oriented InP Core–Shell Nanowire Photovoltaic Devices. *Nano Lett.* **2015**, *15* (3), 1809–1814. https://doi.org/10.1021/nl504559g.

(18) Anttu, N. Absorption of Light in a Single Vertical Nanowire and a Nanowire Array. *Nanotechnology* **2019**, *30* (10), 104004. https://doi.org/10.1088/1361-6528/aafa5c.

(19) Jeyachandran, Y. L.; Mielczarski, E.; Rai, B.; Mielczarski, J. A. Quantitative and Qualitative Evaluation of Adsorption/Desorption of Bovine Serum Albumin on Hydrophilic and Hydrophobic Surfaces. *Langmuir* **2009**, *25* (19), 11614–11620. https://doi.org/10.1021/la901453a.

(20) Verardo, D.; Lindberg, F. W.; Anttu, N.; Niman, C. S.; Lard, M.; Dabkowska, A. P.; Nylander, T.; Månsson, A.; Prinz, C. N.; Linke, H. Nanowires for Biosensing: Lightguiding of Fluorescence as a Function of Diameter and Wavelength. *Nano Lett.* **2018**, *18* (8), 4796–4802. https://doi.org/10.1021/acs.nanolett.8b01360.
